# Efficacy and Safety of Xueshuantong Injection on Acute Cerebral Infarction: Clinical Evidence and GRADE Assessment

**DOI:** 10.3389/fphar.2020.00822

**Published:** 2020-07-02

**Authors:** Jian Lyu, Yanming Xie, Menghua Sun, Lidan Zhang

**Affiliations:** Institute of Basic Research in Clinical Medicine, China Academy of Chinese Medical Sciences, Beijing, China

**Keywords:** Chinese patent medicine, Xueshuantong injection, acute cerebral infarction, randomized controlled trials, meta-analysis, GRADE

## Abstract

**Introduction:**

Xueshuantong injection (XST), a Chinese Medicine, is clinically effective in treating acute cerebral infarction (ACI). However, the meta-analysis of XST combined with conventional treatments (CTs) on ACI remain unexplored. The purpose of this study is to investigate the efficacy and safety of XST combined with CTs on patients with ACI.

**Methods:**

Randomized controlled trials (RCTs) were screened from the Cochrane Library, PubMed, Web of Science, EMBASE, and four Chinese medical databases. The meta-analysis was performed using RevMan 5.3 and STATA 16.0. The GRADE assessment was performed by the GRADEprofiler (GRADEpro version: 3.6). The aggregate 95% confidence intervals (*CI*s) and relative risk (*RR*) estimates were calculated.

**Results:**

Forty studies were included, involving a total of 3,868 patients. XST combined with CTs performed significantly better than CTs alone on the overall response rate (ORR) after treatment (*RR* = 1.21, 95% *CI* = 1.17−1.25, *P* < 0.001). There was no statistical differences in the incidence of adverse reactions between the experimental group (XST plus CTs) and control group (CTs alone). Groups treated with XST substantially decreased the National Institutes of Health Stroke Scale (NIHSS) score compared to the groups without XST (*WMD* = −5.31, 95% *CI =* −6.40 to −4.22, *P* < 0.001). Activities of daily living (ADL) scores were significantly better in the group treated with XST than CTs alone (*WMD =* 12.51, 95% *CI =* 5.6−19.38, *P* < 0.001). Patients who received XST combined with CTs showed significantly higher improvements in high-sensitivity C-reactive protein (hs-CRP) (*WMD* = −2.47, 95% *CI* = −3.11 to −1.82, *P* < 0.001) and interleukin 6 (IL-6) (*WMD* = −13.66, 95% *CI* = −17.80 to −9.51, *P* < 0.001) than those who received CTs alone. The GRADE assessment indicates that the comprehensive quality of this evidence is low.

**Conclusions:**

This meta-analysis and GRADE assessment conditionally recommend that XST combined with CTs can increase the overall response rate, ameliorate neurological deficit, and improve activities of daily living function more than CTs alone. A significant reduction in the hs-CRP and IL-6 levels were observed when XST was combined with CTs.

## Background

Acute cerebral infarction (ACI), also called acute ischemic stroke (AIS), is one of the most common clinical cardiovascular and cerebrovascular diseases (Sun et al., 2015). Due to the high incidence, mortality, and disability rate, ACI has become one of the most serious diseases affecting human health (Arboix and Alio, 2012; Mokin et al., 2014). The neurological deficit syndrome is a pathological basis of ACI, and is caused by necrosis and the softening of brain tissue resulting from ischemia and hypoxia, and its incidence was 60%−70% among all stroke victims (Kim et al., 2012; Hsieh and Chiou, 2014; Atik et al., 2016). In China, 110 out of every 100,000 people suffer from cerebral infarction, accounting for 60-80% of stroke patients (Yong et al., 2013). Overexpressed inflammatory cytokines, such as interleukin 6 (IL-6) and high-sensitivity C-reactive protein (hs-CRP), have a great influence on the occurrence and development of ACI patients (Chen et al., 2018). Infusion of recombinant tissue plasminogen activator (rt-PA) is ACI’s only recognized effective therapy. However, thrombolytic therapy is severely limited by its narrow treatment time windows (< 4.5 hours after onset), fatal adverse events, and high risk of hemorrhagic transformation (Lee et al., 2017). Clinical evidence indicates that only about 2% of ACI patients benefit from thrombolytic therapy (Asahi et al., 2000).

Traditional Chinese Medicines (TCMs) have attracted more and more attention worldwide and have been widely used in clinics in China for more than 3,000 years (Jia et al., 2017; Zhang et al., 2017). In the past few decades, Chinese researchers have been actively investigating more efficient methods of countering ACI using Chinese patent medicine (CPM). CPM contains multiple herbs which are usually prepared by unique methods with a specific combination (Hu and Xu, 2014; Xiao et al., 2014; Ye et al., 2015; Zhong et al., 2015).

Xueshuantong injection (XST) is a standardized herbal preparation, which has been collected by the Pharmacopoeia of the People’s Republic of China and the “2012 national essential drugs list” (Li et al., 2018). XST is generally used for treatment of cerebrovascular diseases, especially ACI (Wang et al., 2015). The results of pharmacological experiments show that XST promotes anticoagulation (Huang et al., 2013), protects endothelial cells (Zhao et al., 2013), reduces leukocyte adhesion, reduces ischemia-reperfusion injury (Li et al., 2009), and improves microcirculation (Lei, 2007).

**Figure 1:**
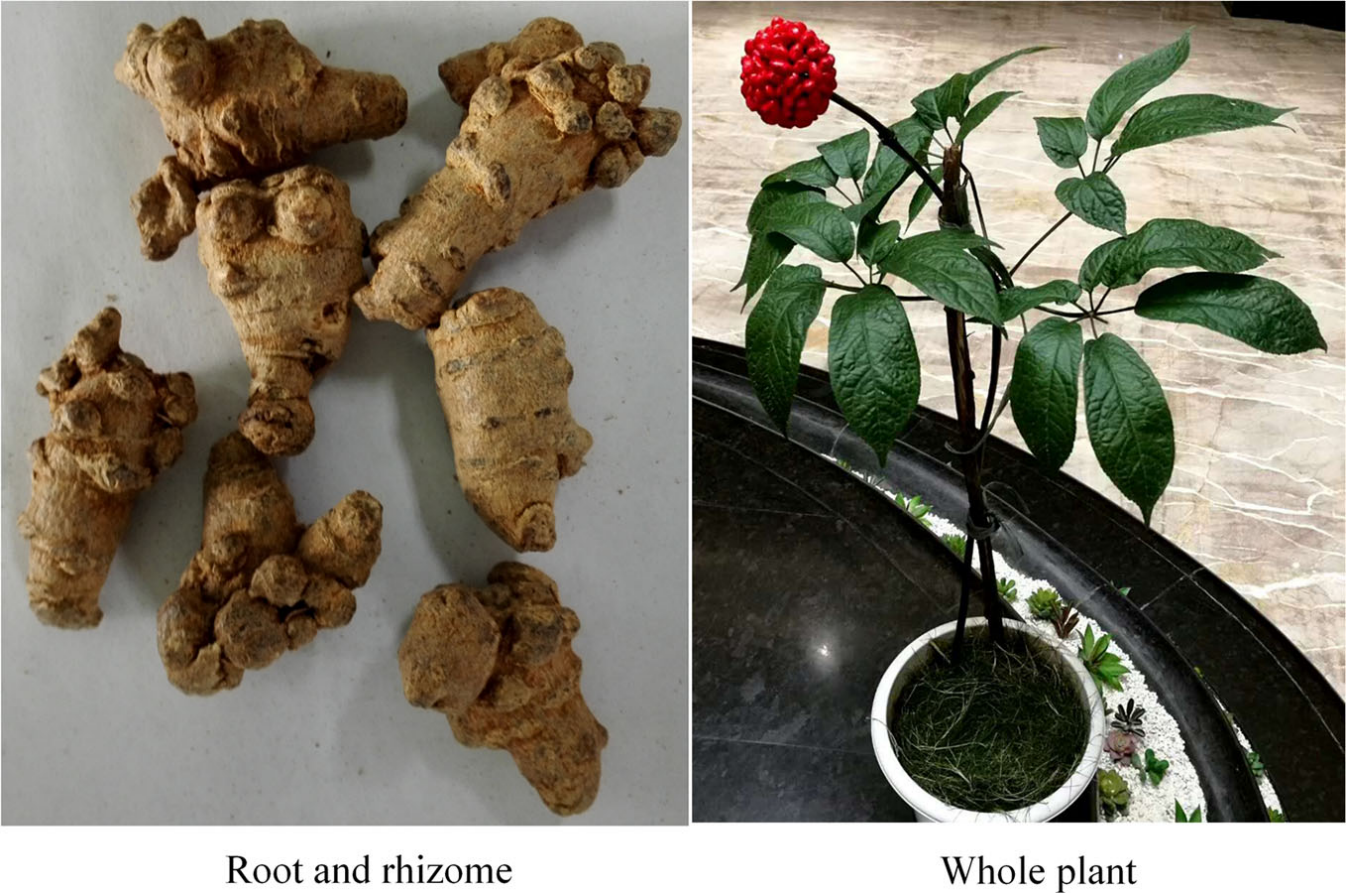
Panax notoginseng.

**Figure 2:**
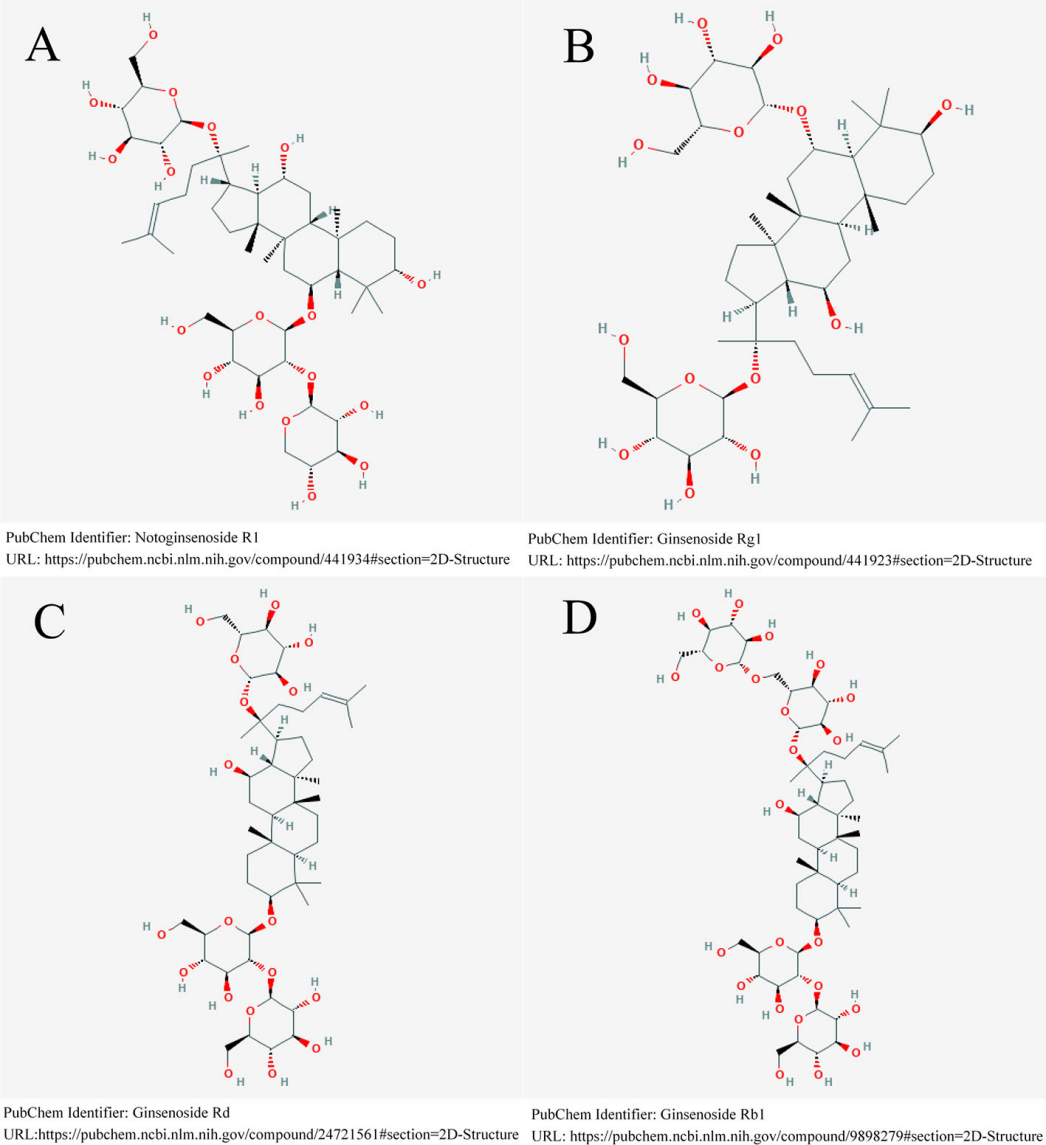
Four main components of PNS. **(A)** notoginsenoside R1. **(B)** ginsenosides Rg1. **(C)** ginsenosides Rd. **(D)** ginsenosides Rb1.

*Panax notoginseng* has been one of the most popular Chinese herbal medicines for hundreds of years, with the functions of removing blood stasis, alleviating pain, relieving swelling, and stopping bleeding (Dong et al., 2003). *Panax notoginseng* saponins (PNS), isolated from the root and rhizome of *Panax notoginseng (Burk.) F. H. Chen* (**Figure 1**), is the main active component of XST (Shen et al., 2017). One study demonstrated that PNS protects against the formation of pathological lesions of cholinergic neurons in a rat model (Zhong et al., 2005). There are four main components of PNS: notoginsenoside R1 (**Figure 2A**; PubChem Identifier: Notoginsenoside R1; URL: https://pubchem.ncbi.nlm.nih.gov/compound/441934#section=2D-Structure), ginsenosides Rg1 (**Figure 2B**; PubChem Identifier: Ginsenoside Rg1; URL: https://pubchem.ncbi.nlm.nih.gov/compound/441923#section=2D-Structure), Rd (**Figure 2C**; PubChem Identifier: Ginsenoside Rd; URL: https://pubchem.ncbi.nlm.nih.gov/compound/24721561#section=2D-Structure), and Rb1 (**Figure 2D**; PubChem Identifier: Ginsenoside Rb1; URL: https://pubchem.ncbi.nlm.nih.gov/compound/9898279#section=2D-Structure) (Toh et al., 2010; Wang et al., 2012; Wang et al., 2013). A previous study has shown that notoginsenoside R_1_ protects the brain from hypoxic-ischemic damage by inhibiting the endoplasmic reticulum (ER) stress pathway (Wang et al., 2016). Ginsenoside Rg1 up-regulates the expression of brain-derived neurotrophic factor (BDNF) in rat brain slices and inhibits tau protein phosphorylation (Li et al., 2012). Evidence from *in vitro* and *in vivo* studies demonstrated that ginsenoside Rd treatment can reduce the infarct volume before and/or after ischemic stroke (Ye et al., 2011a; Ye et al., 2011b; Ye et al., 2011c), increase neuronal survival (Ye et al., 2009; Li et al., 2010; Hu et al., 2013; Zhang et al., 2013), and enhance cognitive and neurological functions (Ye et al., 2011a; Ye et al., 2011c; Zhang et al., 2014). More information is shown in **Supplementary Table S1**.

Over the past decade, China has conducted a large number of clinical trials to explore the efficacy and safety of XST combined with conventional therapy (CTs) for patients with ACI. The objective of this study was to evaluate the efficacy and safety of XST combined with CTs for patients with ACI and grade the level of evidence with the Grading of Recommendations Assessment, Development and Evaluation (GRADE) system. According to our knowledge, this is the first meta-analysis and GRADE assessment focusing on the combination of XST and CTs for the efficacy and safety of ACI. The clinical evidence obtained from this study can be used to assist clinicians in choosing between XST combined with CTs and CTs alone in daily practice.

## Methods

This meta-analysis was conducted and reported according the PRISMA statement (Liberati et al., 2009). The study protocol was registered with PROSPERO (registration number: CRD42020164548).

### Eligibility Criteria

#### Types of Studies

Randomized controlled trials (RCTs) in which XST combined with CTs (experimental group) was compared with CTs (control group), where there was at least 14 days of treatment period from randomization. Studies were excluded in the case of duplicate studies, non-RCT design, no measured outcomes of interest, or interventions with TCMs (extract injections, oral medicine, decoctions, acupuncture, and massage). Types of Participants: adults over 18 years old diagnosed with ACI or AIS.

#### Types of Interventions

An experimental group treated with XST plus CT ands a control group treated with CTs. CTs including thrombolytic drugs, anticoagulant, antiplatelet, antihypertensive, statins, neuroprotective agents, collateral circulation drugs, and lipid-lowering medications were considered.

#### Types of Outcomes

Efficacy outcomes, including overall response rate (ORR), NIHSS score, Activities of daily living (ADL) score, and hs-CRP and IL-6 levels. Safety outcomes included incidences of adverse reactions and adverse events.

### Search Strategy

PubMed, Embase, Web of Science, Cochrane Library, China National Knowledge Infrastructure (CNKI), VIP Chinese Sci-tech Periodical Database (VIP), China Biological Medicine Database (CBM), and Chinese Wan Fang Database (Wan fang) were searched from inception of the database to July 2018. The search strategies for PubMed are presented in **Supplementary Table S2**. This search strategy includes all search terms, and other electronic databases will be modified as required.

### Study Selection

The guidelines provided in the PRISMA statement were followed during the study selection process (Liberati et al., 2009). JL and MHS independently performed all literature selection according to the predefined study selection eligibility. The records retrieved in all databases were imported into Endnote 9.0 software, and the duplicated parts were removed. Based on the information in the title and abstract, the electronic database was initially screened for qualified studies. Then, by reading the full text, the selected studies were screened to decide whether to include it or not, and the disagreements in the screening process were resolved by YMX.

### Data Extraction

We performed data extraction with reference to Cochrane Collaboration’s inclusion study data extraction template (Cochrane Consumers and Communication La Trobe University et al., 2018). JL and LDZ were responsible for the extraction of relevant data from each eligible RCT and for placing it into an electronic data-extraction sheet (Microsoft Excel). The characteristics of the included studies were extracted, and the completeness and consistency among the studies were compared by the investigators. If there was a disagreement between the two investigators, YMX was consulted for the final consensus.

### Data Items

Data extracted included: (1) number of events and sample sizes in each group, (2) mean/standard deviation (SD) of each group, (3) the first author, (4) year of publication, (5) study design, (6) specific method of randomization and blinding, (7) age and gender of the two groups, (8) intervention/dosage/course of treatment in each group, (9) outcomes, and (10) adverse events.

### Risk of Bias Assessment

The Cochrane risk of bias (ROB) assessment tool was used to assess the bias assessment risk of all eligible studies (Higgins et al., 2011). Each study was evaluated from the following seven aspects: random sequence generation, allocation concealment, blinding of participants and personnel, blinding of outcome assessors, incomplete outcome data, selective outcome reporting, and other biases. The ROB was judged as “low”, “high”, or “unclear”. The investigators resolved disagreements through discussion to reach consensus.

### Data Analysis

Review Manager Version 5.3 and Stata 16.0 were used for the data analysis and synthesis. The data on the outcomes were combined, and the effect sizes were expressed as weighted mean difference (*WMD*) and relative risk (*RR*) with 95% confidence intervals (*CIs*). The heterogeneity among studies was identified if the *I^2^* was greater than or equal to 50% or *P* value was less than or equal to 0.10, and a random-effects model was used; if the *I^2^* statistic was under 50% or *P* value was greater than 0.10, a fixed-effects model was used (Higgins and Thompson, 2002; Higgins et al., 2003). A sensitivity analysis was performed to explore potential sources of heterogeneity. Begg’s test and Egger’s linear regression test were used for publication bias, and we considered *P* < 0.05 to be significant. Stabilities of synthetic results were evaluated with a visual assessment of sensitivity analyses. The method of omitting each study in sequence was used for sensitivity analysis. The meta-analysis followed the standard PRISMA checklist (see **Supplementary files**).

### Assessment of Evidence Quality

The assessment of evidence quality was performed by JL and YX independently according to the GRADE criteria (Balshem et al., 2011; Guyatt et al., 2011a; Guyatt et al., 2011b). The evidence quality of included outcomes was graded as high, moderate, low, or very low. RCTs were initially classified as having high-quality evidence. The quality of each outcome was downgraded for the following five factors: risk of bias, inconsistency, indirectness, imprecision, and publication bias. GRADEpro3.6.1 software was used for the data analysis and synthesis.

## Results

### Study Selection

**Figure 3:**
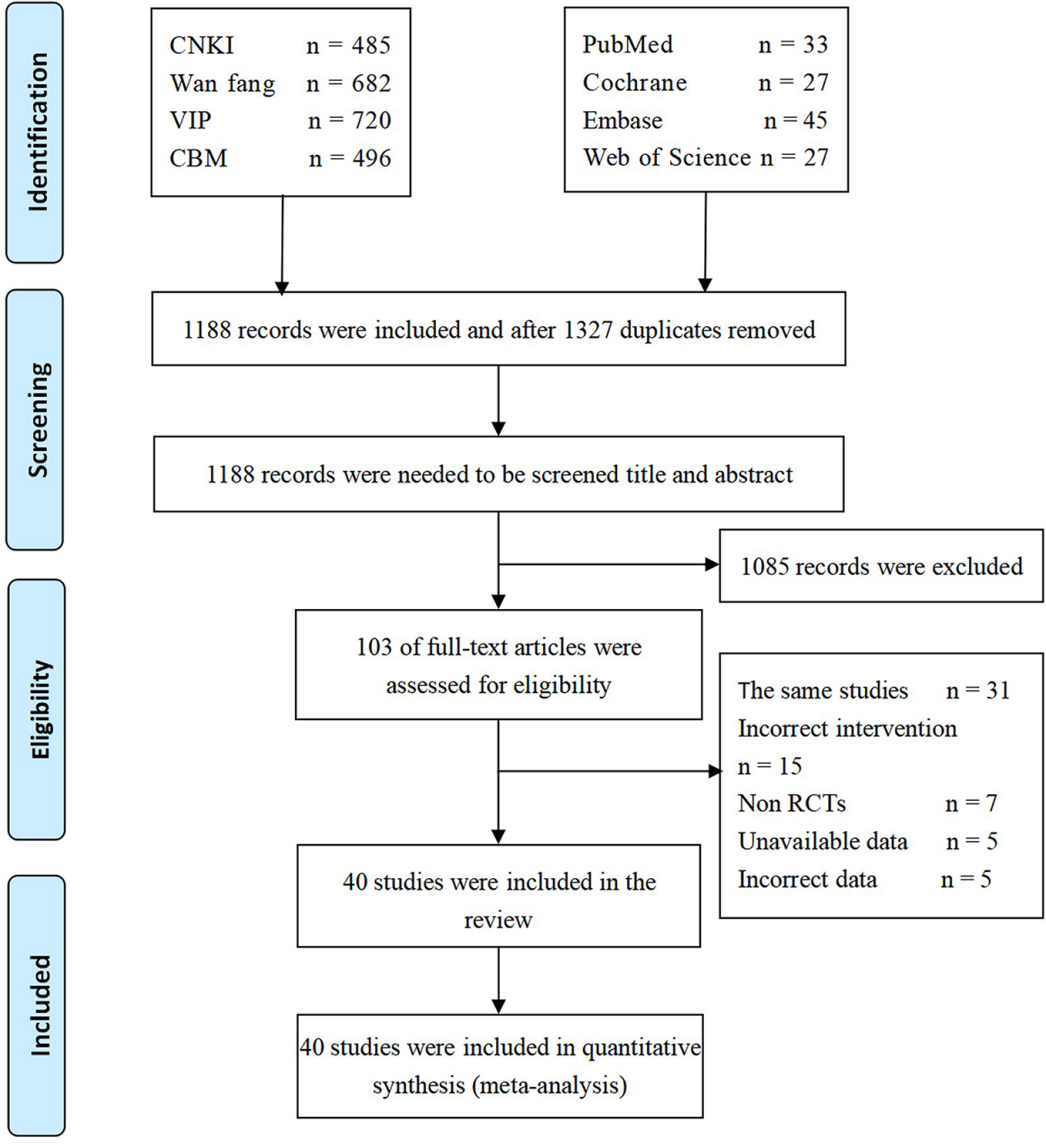
PRISMA flow diagram of included and excluded articles. PRISMA, Preferred Reporting Items for Systematic Reviews and Meta-Analysis.

The final review included 40 studies (Yang et al., 2004; Wang et al., 2009; Wang, 2009; Gao, 2010; Ma, 2010; Zhao, 2010; Li, 2011; Sun, 2011; Wang, 2011; Duan and Shang, 2012; Liang, 2012; Li, 2012a; Li, 2012b; Zheng, 2012; Gong and Gong, 2013; Wang, 2013; Zhang, 2013; Li, 2014; Wang, 2014; Zhang, 2014; Chen, 2015; He et al., 2015; Li, 2015; Liu, 2015; Yang and Wei, 2015; Jiao et al., 2016; Zhang et al., 2016; Zheng, 2016; Gu and Yang, 2017; Shu, 2017; Chen, 2018; Chen and Ding, 2018; Ren et al., 2018; Wang et al., 2018; Du, 2019; Li, 2019; Liu and Wang, 2019; Xu et al., 2019; Zhang et al., 2019a; Zhang et al., 2019b), and all were published in Chinese. The PRISMA flow diagram of included and excluded articles is shown in **Figure 3**.

### Study and Participant Characteristics

A total of 40 studies were included, which involved 3,868 patients. Experimental groups included 1,994 patients, and control groups included 1,913 patients, all of which described the comparability of the baselines between two groups. The interventions of the 40 studies are XST combined with CTs versus CTs. The details are shown in **Supplementary Table S3**.

### Methodological Quality

**Figure 4:**
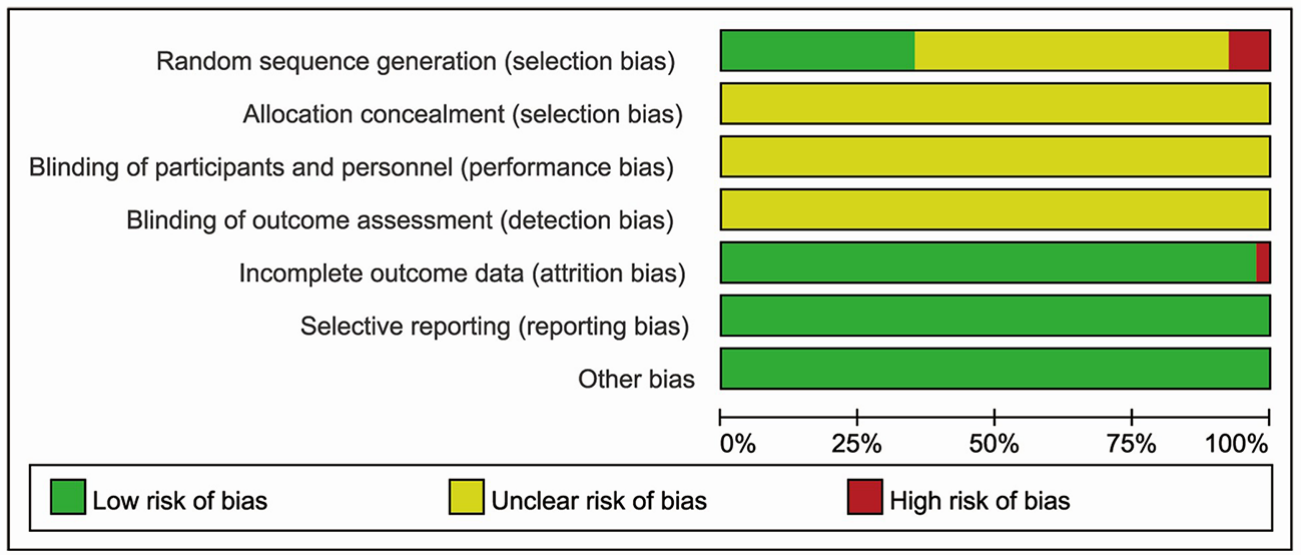
Risk of bias graph reviewing authors’ judgments about each risk of bias item presented as percentages across all included studies.

**Figure 5:**
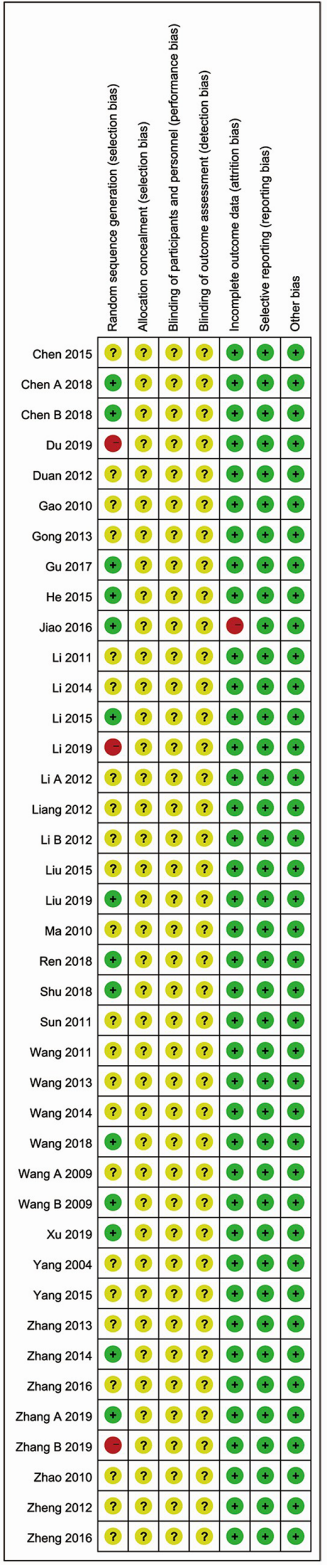
Risk of bias summary reviewing authors’ judgments about each risk of bias item for each included study.

No study described the protocol and sample size calculation. As for generation of random sequence, thirteen studies used specific methods, including random number tables (Jiao et al., 2016; Gu and Yang, 2017; Shu, 2017; Chen and Ding, 2018; Liu and Wang, 2019; Xu et al., 2019; Zhang et al., 2019a), random in order of visits (He et al., 2015; Li, 2015; Wang et al., 2018), coin flipping (Chen, 2018), the picking method (Ren et al., 2018), and SAS12.0 software (Zhang, 2014). Details of allocation concealment and study blinding were not adequately reported. One study had the high risk of “incomplete data” (Jiao et al., 2016). All studies were considered to be free of “selective outcomes reporting”. No other baseline characteristics (age, sex, severity of acute cerebral infarction) were significantly different between the two groups. There were no other potential sources of bias in all included trials. The bias risk assessment is shown in **Figures 4** and **5**.

### Efficacy Outcomes

#### Overall Response Rate

**Figure 6:**
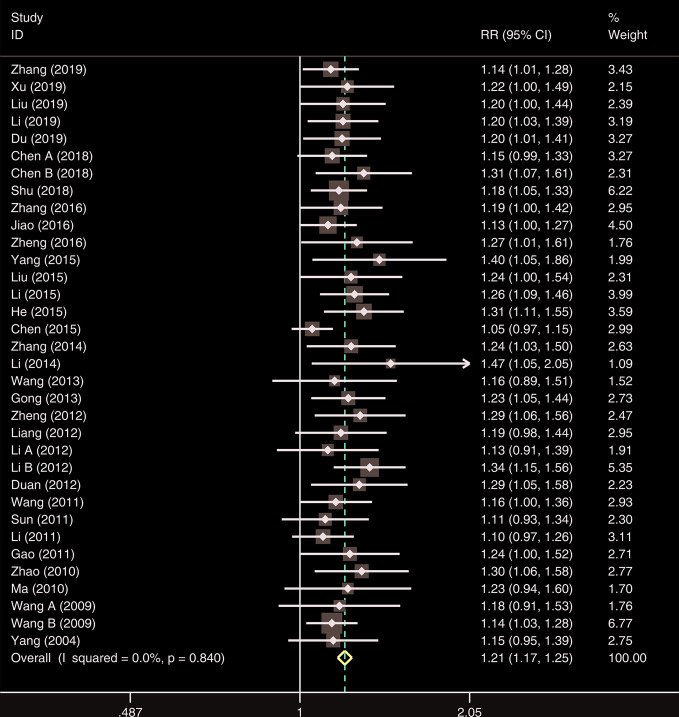
The forest plot reflects the efficacy of XST plus CTs vs CTs alone on overall response rate. XST, Xueshuantong injections; CTs, conventional treatments; RR, relative risk; CI, confidence interval.

A total of thirty-four studies were analyzed for the overall response rate. The heterogeneity among all studies was insignificant (*I²*= 0.0%, *P* = 0.840), and a fixed-effects model was used. The results demonstrated that the overall response rate for the observation group (XST plus CTs) was significantly higher than that of CTs alone (*RR* = 1.21, 95% *CI* = 1.17−1.25, *P* < 0.001; **Figure 6**).

#### NIHSS Score

**Figure 7:**
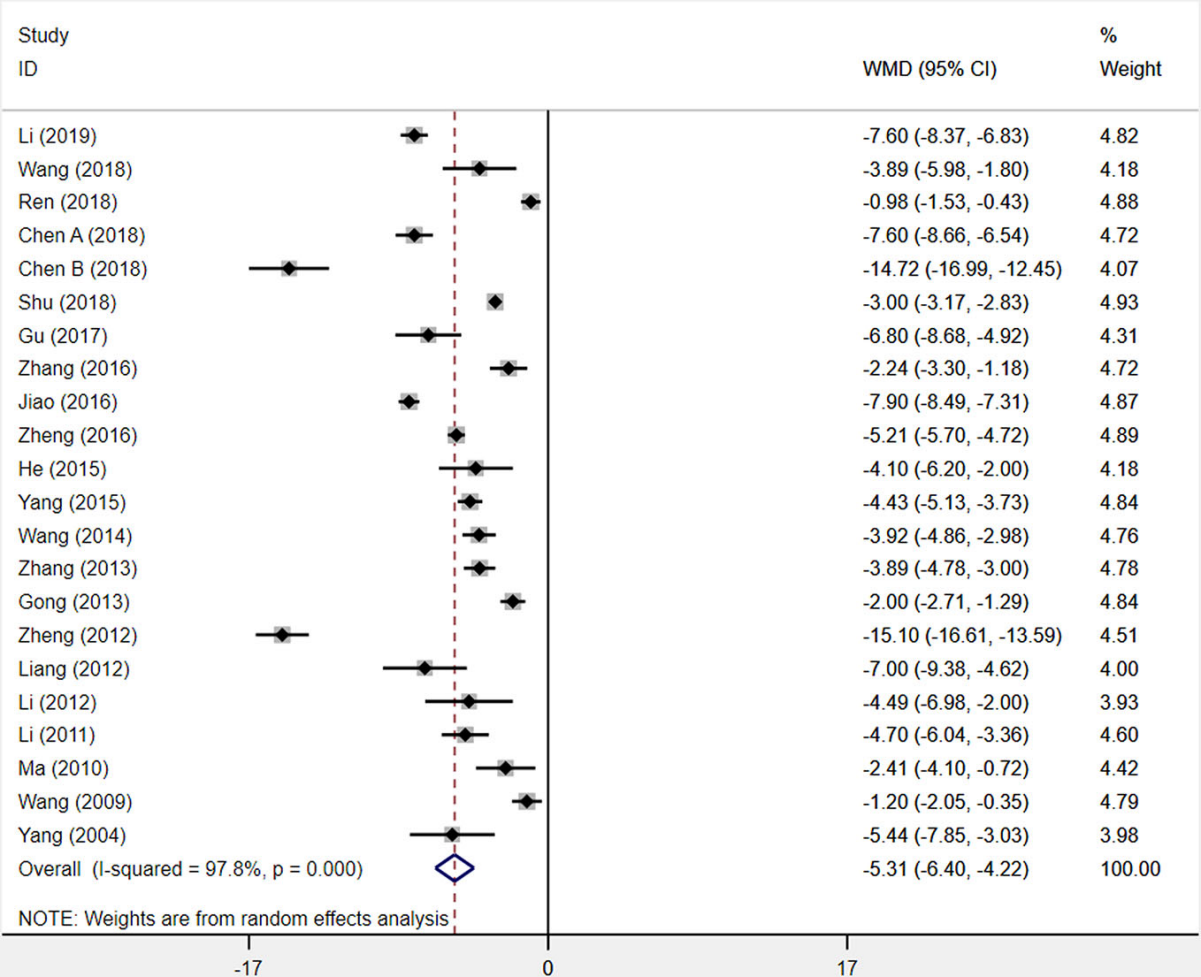
The forest plot reflects the efficacy of XST plus CTs vs CTs alone on the NIHSS score. XST, Xueshuantong injections; CTs, conventional treatments; WMD, weighted mean difference; CI, confidence interval.

Analysis used a random-effects model of twenty-two included trials comparing XST combined with CTs vs. CTs only, and found that XST combined with CTs was related to a significant reduction in NIHSS score (*WMD* = −5.31, 95% *CI* = −6.40 to −4.22, *P* < 0.001; **Figure 7**). Nevertheless, the heterogeneity among the twenty-two studies was statistically significant (*I²* = 97.8%, *P* < 0.001).

Sensitivity analysis was carried out to explore the sources of heterogeneity, but these sources were not identified. From the twenty-two included studies, we observed that the differences of dosage, duration, and diversiform regimens of CTs among studies may be responsible for this heterogeneity but have not been investigated. This result was limited by substantial heterogeneity. More trials with good homogeneity would be needed to prove the results.

#### ADL Score

**Figure 8:**
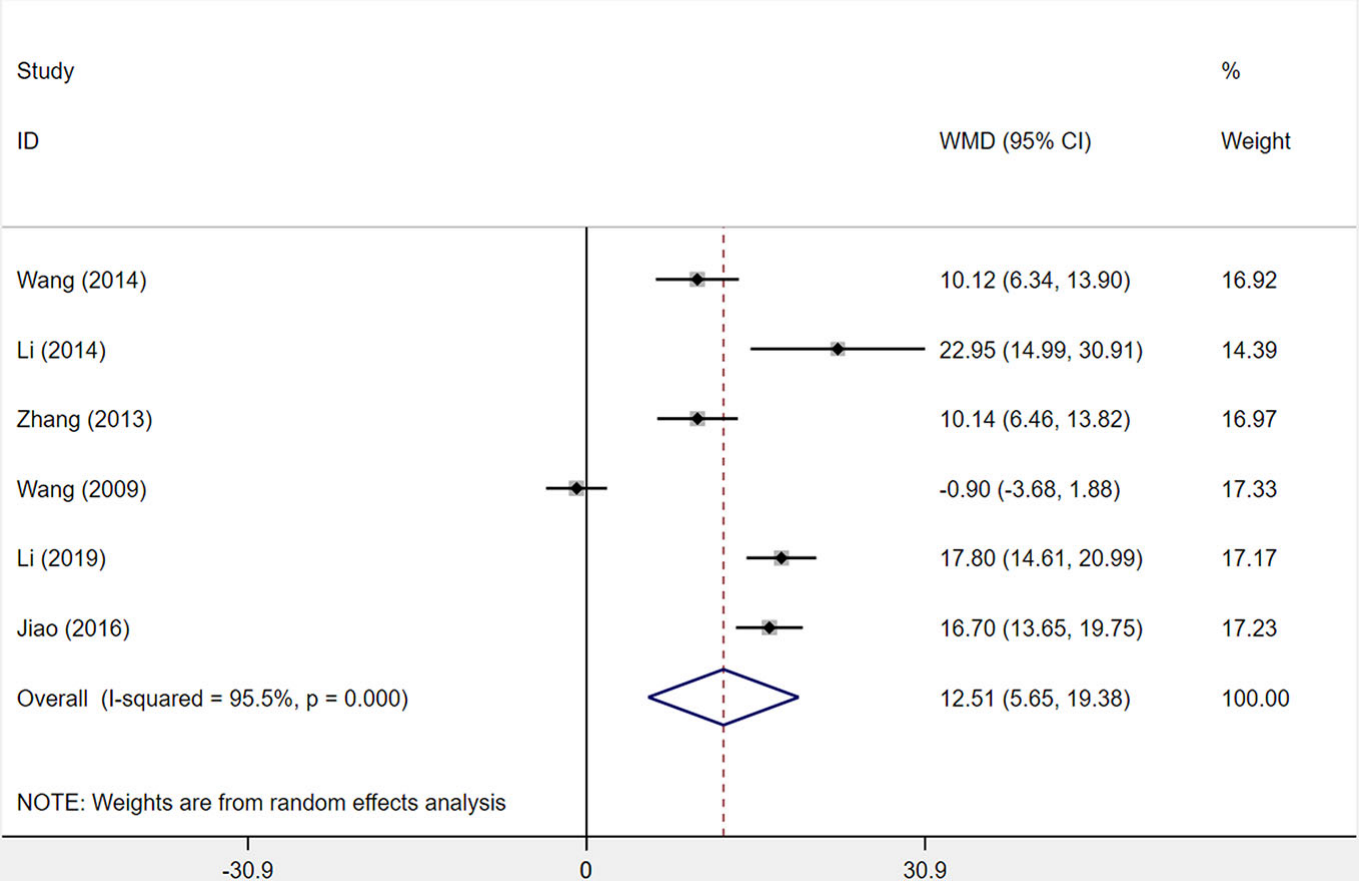
The forest plot reflects the efficacy of XST plus CTs vs CTs alone on the activities of daily living score. XST, Xueshuantong injections; CTs, conventional treatments; WMD, weighted mean difference; CI, confidence interval.

Six studies were included to assess changes in the activities of daily living (ADL) score. Among the six studies comparing XST combined with CTs vs. CTs alone, there was a consequential difference in the ADL score across six studies: treatment with XST combined with CTs, as compared with CTs independently, was associated with a momentous improvement in ADL score (*WMD* = −12.51, 95% *CI* = −5.65 to −19.38, *P* < 0.001; **Figure 8**). However, the significant heterogeneity was identified among the studies (*I^2^* = 95.5%, *P* < 0.001).

Sensitivity analysis and reading the original literature found that the dosage and duration of treatment in four studies (Wang et al., 2009; Li, 2014; Jiao et al., 2016; Li, 2019) were quite different from those of two other studies, which were the major source of the heterogeneity. We removed the four studies and pooled the other two studies alone (*WMD* = 10.13, 95% *CI* = 7.49−12.77, *P* < 0.001; **Supplementary Figure S1**). Heterogeneity between two studies was insignificant (*I²* = 0%, *P* = 0.994).

#### Hs-CRP

**Figure 9:**
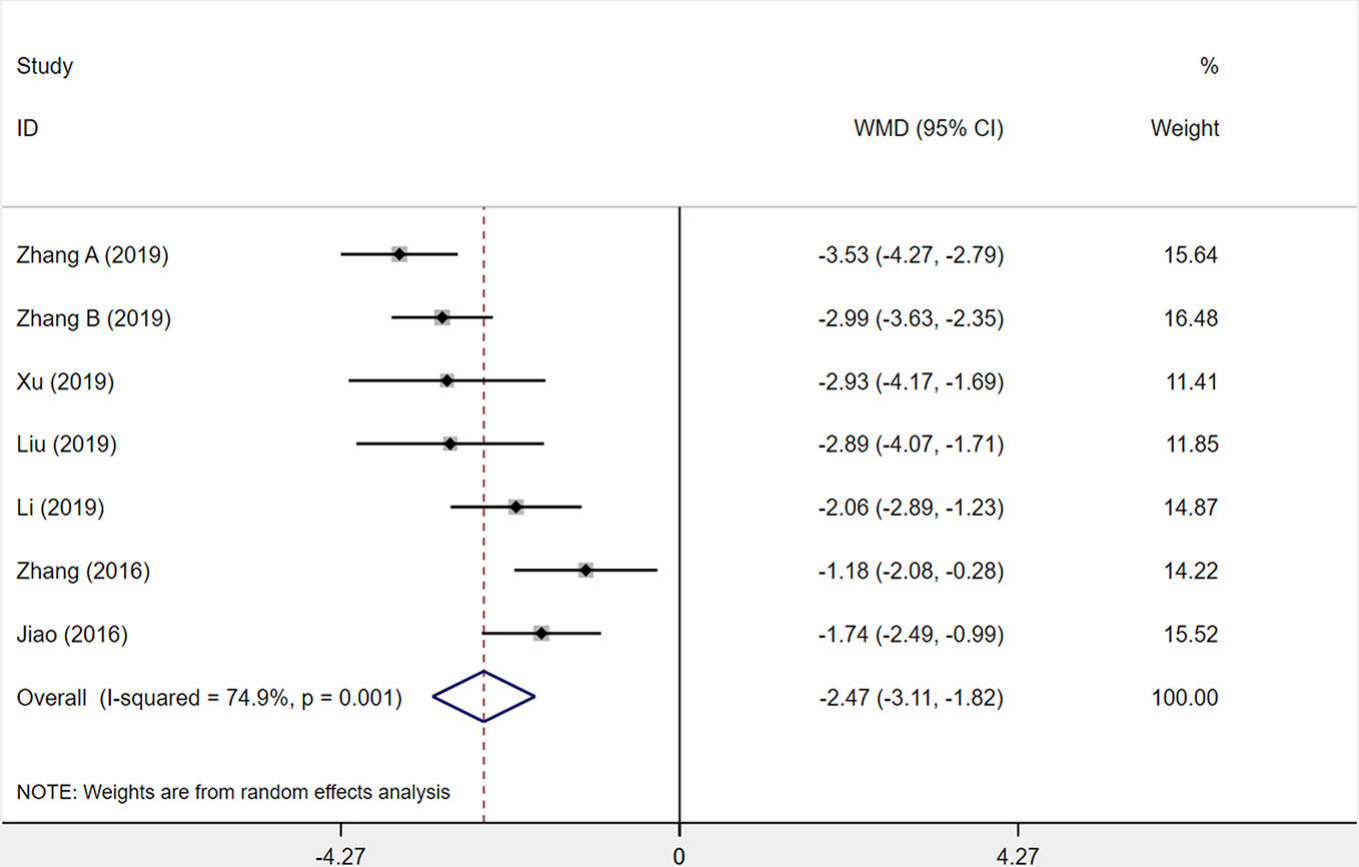
The forest plot reflects the efficacy of XST plus CTs vs CTs alone on hs-CRP levels. XST, Xueshuantong injections; CTs, conventional treatments; WMD, weighted mean difference; CI, confidence interval.

Seven studies were assessed for the serum level of hs-CRP after patients had been treated. The results demonstrated that XST combined with CTs showed a weighty decrease on the hs-CRP level among seven studies compared with CTs alone (*WMD* = −2.47, 95% *CI* = −3.11 to −1.82, *P* < 0.001; **Figure 9**). However, significant heterogeneity was identified among the studies (*I^2^* = 74.9%, *P* = 0.001).

In the forest plot, three trials’ (Jiao et al., 2016; Zhang et al., 2016; Zhang et al., 2019a) confidence intervals did not overlap with those of the other four trials. We undertook sensitivity analysis and read the original literature to explore the sources of heterogeneity and found that the treatment dosages of these three studies were quite different from those of the other studies. We removed the three studies and pooled the other four studies alone (*WMD* = −2.71, 95% *CI* = −3.15 to −2.28, *P* < 0.001; **Supplementary Figure S2**). Heterogeneity among the four studies was insignificant (*I²* = 10%, *P* = 0.343).

#### IL-6

**Figure 10:**
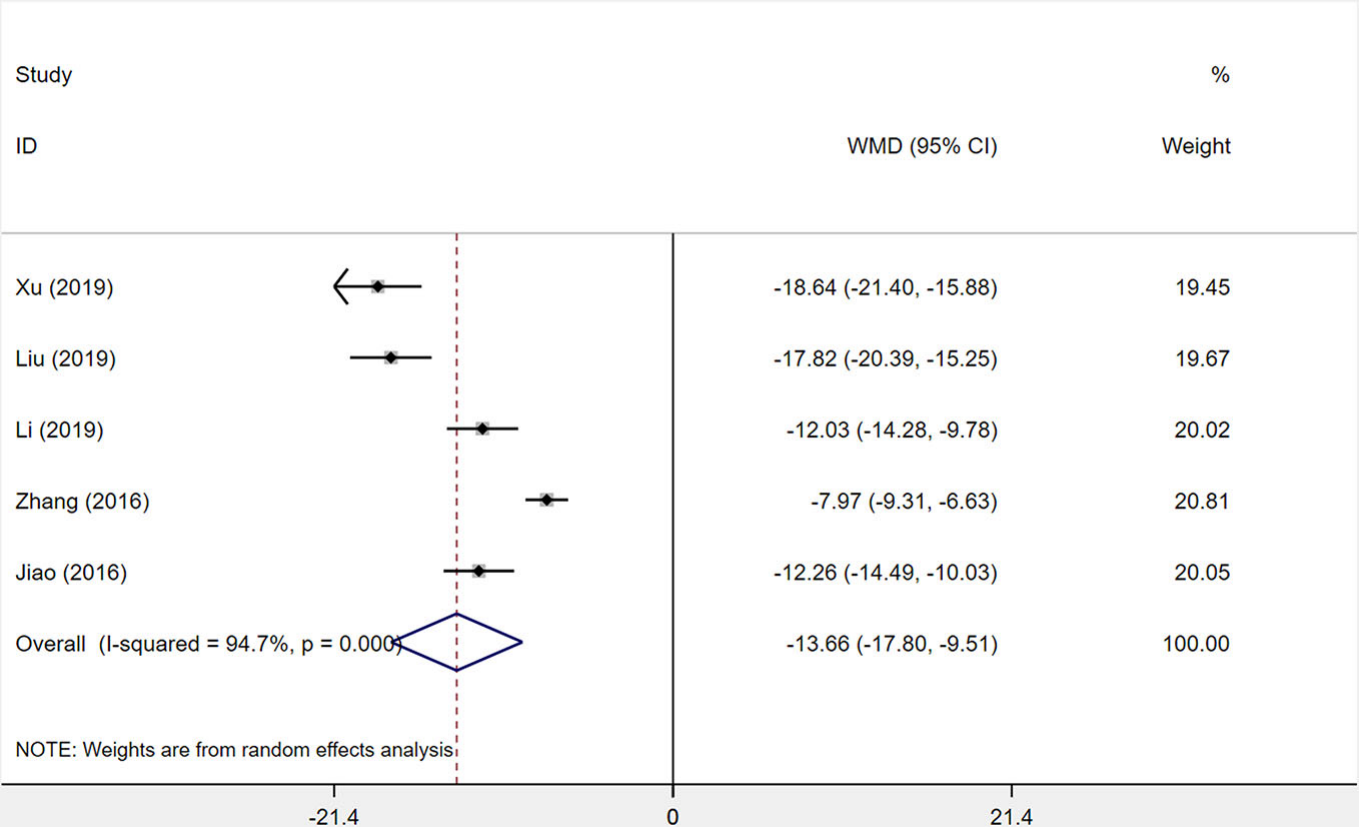
The forest plot reflects the efficacy of XST plus CTs vs CTs alone on IL-6 levels. XST, Xueshuantong injections; CTs, conventional treatments; WMD, weighted mean difference; CI, confidence interval.

A total of five studies was assessed for the level of interleukin-6 (IL-6) after treatment with XST plus CTs compared with CTs alone. The heterogeneity among the five studies was statistically relevant (*I^2^* = 94.7%, *P* < 0.001), and a random-effects model was used. The XST combined with CTs showed a consequential decrease at the IL-6 level among the five studies compared with CTs alone (*WMD* = −13.66, 95% *CI* = −17.80 to −9.51, *P* < 0.001; **Figure 10**).

After sensitivity analysis and careful reading of the original literature, we found that the treatment dosage of three studies (Zhang et al., 2016; Liu and Wang, 2019; Xu et al., 2019) were quite different from those of two other studies, which were the major source of the heterogeneity. We removed the three studies and pooled the other two studies alone (*WMD* = −12.15, 95% *CI* = −13.73 to −10.56, *P* < 0.001; **Supplementary Figure S3**). Heterogeneity between the two studies was insignificant (*I^2^* = 0%, *P* = 0.887).

### Safety Outcomes

#### Incidence of Adverse Reactions

**Figure 11:**
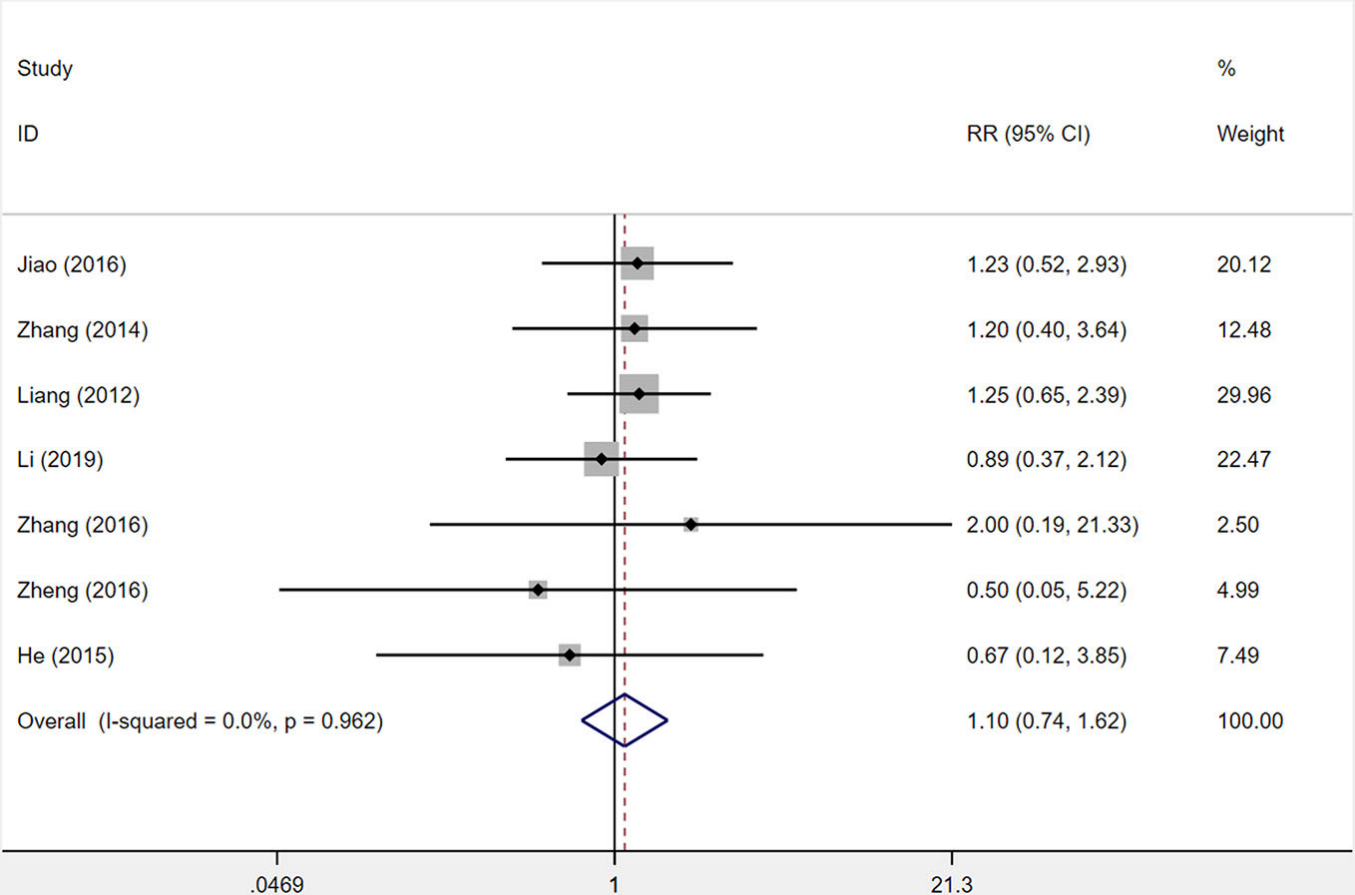
The forest plot reflects the safety of XST plus CTs vs CTs alone on the incidence of adverse reactions. Abbreviation: XST, Xueshuantong injections; CTs, conventional treatments; RR, relative risk; CI, confidence interval.

Seven studies were included to determine the incidence of adverse reactions. Incidence of adverse reactions occurred in 44 out of 352 patients (12.5%) who received XST combined with CTs and 40 out of 351 patients (11.4%) who received CTs alone. The heterogeneity among seven studies was insignificant (*I^2^* = 0.0%, *P* = 0.962), and a fixed-effects model was used. No statistical difference was observed between XST combined with CTs and CTs alone (*RR* = 1.10, 95% *CI* = 0.74−1.62, *P* = 0.643; **Figure 11**).

#### Adverse Events

Eighteen studies reported adverse events, and twenty-three of these studies reported positive results. Among them, eleven studies reported no adverse events in both groups (Wang, 2009; Gao, 2010; Ma, 2010; Li, 2011; Wang, 2011; Duan and Shang, 2012; Gong and Gong, 2013; Zhang, 2013; Li, 2015; Liu, 2015; Yang and Wei, 2015). Seven studies (Liang, 2012; Zhang, 2014; He et al., 2015; Jiao et al., 2016; Zhang et al., 2016; Zheng, 2016; Li, 2019) reported adverse events and abnormal laboratory indices in two groups including headache, dizziness, gastrointestinal discomfort, elevation of transaminase, and other symptoms. Site investigators determined that these adverse events were not caused by the study drug. No participants discontinued the study drug due to adverse events. The details are shown in **Supplementary Table S4**.

#### Publication Bias

**Figure 12:**
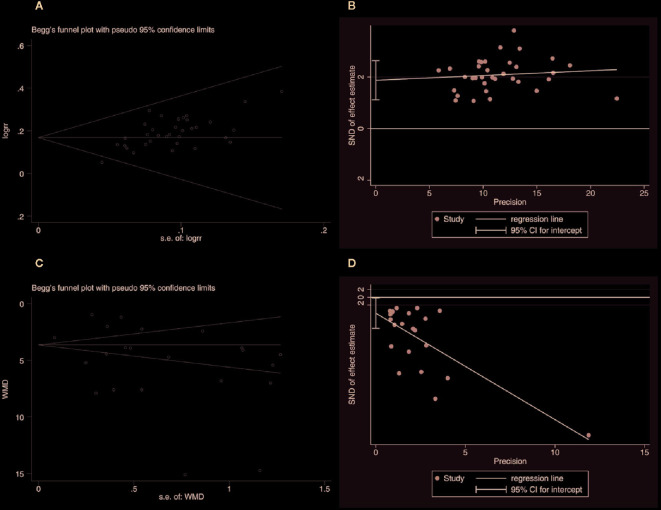
Publication bias. **(A)** The Begg’s test on overall response rate. **(B)** The Egger’s linear regression test on overall response rate. **(C)** The Begg’s test on the NIHSS score. **(D)** The Egger’s linear regression test on the NIHSS score.

In order to assess the publication bias of the overall response rate and NIHSS score, Begg’s test and Egger’s linear regression test were conducted. The Begg’s test (**Figure 12A**) and Egger’s linear regression test (**Figure 12B**) of the overall response rate demonstrated that the *P* value was all less than 0.05 (Begg, *P* = 0.006; Egger, *P* < 0.001), which indicates that there is publication bias. The Begg’s test (**Figure 12C**) of the NIHSS score demonstrated that the *P* value was greater than 0.05 (*P* = 0.367), but the Egger’s linear regression test (**Figure 12D**) demonstrated that the *P* value was less than 0.05 (*P* = 0.041). Considering that the efficiency of Egger’s linear regression test is more accurate than Begg’s test, we could not rule out publication bias of the NIHSS score.

The potential publication bias might be due to the following four items: the small sample size of the included studies brings a small sample effect; significant clinical and statistical heterogeneity; the positive results are easy to publish in China; and all the included studies are in Chinese, which might lead to language publication bias. The above test results of publication bias still need to be further verified by larger samples and rigorous RCTs.

#### Sensitivity Analysis

The sensitivity analysis was carried out for six outcomes. Sensitivity analysis was performed by omitting individual studies one by one to assess their effect on the aggregated results. Since there was no change in the statistical significance of the results when any single study was omitted, this indicates the reliability of the results. Results are shown in **Supplementary Figure S4** (**Figure S4A**: overall response rate; **Figure S4B**: incidence of adverse reactions; **Figure S4C**: NIHSS score; **Figure S4D**: ADL score; **Figure S4E**: hs-CRP; **Figure S4F**: IL-6).

#### GRADE Assessment

The GRADE system was used to assess the level of evidence for the six outcomes, which indicated low or very low quality with serious methodological problems (risk of bias and reporting bias) and a heterogeneity problem (indirectness). The GRADE evidence profiles are shown in **Table 1**.

**Table 1:** GRADE evidence profile.

Quality assessment						No of patients		*RR*/*WMD* (95% *CI*)	*P* value	Quality
No. of studies	Risk of bias	Inconsistency	Indirectness	Imprecision	Other considerations	XST combined with CTs	CTs			
**Overall response rate**										
34	serious^a,b,c,d,^	no serious inconsistency	no serious indirectness	no serious imprecision	reporting bias^e^	1560/1682 (92.7%)	1232/1607 (76.7%)	*RR* = 1.21 (1.17−1.25)	<0.001	⊕⊕OO low
**Incidence of adverse reactions**										
7	serious^a,b,c^	no serious inconsistency	no serious indirectness	no serious imprecision	reporting bias^e^	44/352 (12.5%)	40/351 (11.4%)	*RR* = 1.10 (0.74−1.62)	= 0.643	⊕⊕OO low
**NIHSS score**										
22	serious^a,b,c,d^	serious^f^	no serious indirectness	no serious inconsistency	reporting bias^e^	1035	985	*WMD* = −5.31 (−6.40 to −4.22)	<0.001	⊕OOO very low
**Activities of daily living score**										
6	serious^a,b,c,d^	serious^f^	no serious indirectness	no serious inconsistency	reporting bias^e^	253	249	*WMD* = 12.51 (5.65 −19.38)	<0.001	⊕OOO very low
**High sensitivity C-reactive protein**										
7	serious^a,b,c,d^	no serious inconsistency	no serious indirectness	no serious imprecision	reporting bias^e^	398	397	*WMD* = −2.47 (−3.11 to −1.82)	<0.001	⊕⊕OO low
**Interleukin-6**										
5	serious^a,b,c,d^	serious^f^	no serious indirectness	no serious inconsistency	reporting bias^e^	239	238	*WMD* = −13.66 (−17.80 to −9.51)	<0.001	⊕OOO very low

XST, Xueshuantong injection; CTs, conventional treatment; CI, Confidence interval; RR, relative risks; WMD, weighted mean difference; ^a^No details of random protocol were reported; ^b^Lack of allocation concealment; ^c^Didn’t report the implementation of blinding; ^d^Accounting for the patients and the outcome events is not complete; ^e^Quantitative evaluation of the included data indicated publication bias; ^f^I^2^^ ^> 90% for heterogeneity.

## Discussion

The primary efficacy endpoint of the present study was the overall response rate (ORR). ORR refers to the sum of the percentage of patients who achieve complete or partial responses (FDA, 2007). ORR criterion is the sum of the percentage of patients whose NIHSS score decreased by 91% to 100%, 46% to 90%, or 18% to 45%. The overall response rate in patients who had been treated with XST plus CTs was 92.7% (1560/1682). The overall response rate in patients who had been treated with CTs independently was 76.7% (1232/1607). The results indicated that the ORR of XST combined with CTs was remarkably higher than CTs alone. The secondary efficacy endpoints were the NIHSS score, ADL score, and hs-CRP and IL-6 levels. The level of the NIHSS score can reflect the clinical situation of the patient with ACI more intuitively. The NIHSS score reveals the extent of neurological damage and is usually used to evaluate the efficacy of patients with ACI after therapy (Wu et al., 2019). In this study, twenty-two studies with 2020 patients were assessed using the NIHSS score. The result concluded that the NIHSS score of the experimental group was significantly lower in patients with XST plus CTs compared to the control group (CTs). Nevertheless, the result was limited by unidentified heterogeneity. With the limitation of substantial heterogeneity, it is necessary to increase the sample size to verify whether the use of XST during the acute stage of ACI really decreases the NIHSS score.

In the present study, the ADL score was used to comprehensively assess quality of life following ACI. Six studies with 502 patients were observed using the ADL score, in which the experimental group (XST plus CTs) had a higher ADL score than the control group (CTs). The substantial heterogeneity of the ADL score was identified. Four studies with different dosage and duration of treatments were the major source of the heterogeneity. After the above four studies were removed, heterogeneity had no clinical significance (*I²* = 0%) and the statistical result was not changed (*P* < 0.001). Results indicated that the addition of XST combined with CTs may significantly improve the activities of daily living for patients with ACI. Literature studies have found that overexpressed inflammatory cytokines play an important role in the evaluation of the therapeutic efficacy of ACI patients (Chen et al., 2018). The Low-grade inflammation hs-CRP and proinflammatory cytokine IL-6 levels simultaneously significantly affect the pathophysiological process of ACI brain cells (Moreno et al., 2004; Segers et al., 2007). In this study, a total of 795 patients in seven studies were assessed for their levels of hs-CRP, and 577 patients in five studies were assessed for their levels of IL-6. We observed significantly lower mean levels of IL-6 and hs-CRP among experimental groups (XST plus CTs) compared with control groups (CTs), but the heterogeneities were identified. The sources of heterogeneity have been explored through sensitivity analysis, and the statistical results were not changed after removing the studies that resulted in heterogeneity. The results showed that using XST combined with CTs can significantly reduce the levels of IL-6 and hs-CRP, which provides an objective reference for the efficacy of XST for patients with ACI.

The primary safety endpoint of the present study was the incidence of adverse reactions. Seven studies with 703 patients were observed for incidence of adverse reactions. The incidence of adverse reactions in patients who had been treated with XST plus CTs was 12.5% (44/352). The incidence of adverse reactions in patients who had been treated with CTs solely was 11.4% (40/351). The results indicated that the incidence of adverse reactions in the experimental group (XST plus CTs) was not statistically different from that in the control group (CTs only), which suggested that adding the use of XST combined with CTs was still safe. The secondary safety endpoint was adverse events. Eighteen studies with 1612 patients were observed for adverse events. In the present study, eleven studies with 909 patients reported no adverse events in both groups. Mild adverse events were observed in seven studies and mainly consisted of headache, dizziness, gastrointestinal discomfort, elevation of transaminase, and other unreported symptoms. One study (Li, 2019) reported that minor gastrointestinal reactions (n=2), elevation of transaminase (n=2), and gingival bleeding (n=4) occurred in the experimental group; one study (Zhang et al., 2016) reported that abnormal liver function (n=2) and mild headache (n=1) occurred in the experimental group; one study (Jiao et al., 2016) reported that minor gastrointestinal reactions (n=4), elevation of transaminase (n=3), and gingival bleeding (n=3) occurred in the experimental group; and one study (Zheng, 2016) reported that one patient occurred without the specific symptoms reported. All the above adverse events were tolerable and did not affect treatment. One study (He et al., 2015) reported that facial fever occurred in two patients, and it completely disappeared after a proper rest. One study (Zhang, 2014) reported that headache (n=3), fever (n=2), and bleeding (n=1) occurred in the experimental group, and the symptoms disappeared after symptomatic treatment. One study (Liang, 2012) reported that fifteen patients occurred without the specific symptoms reported in the experimental group, and the symptoms were tolerable and did not affect treatment. No significant safety issues happened, and all adverse events were considered mild and unrelated to the study drug. However, due to problems of combined drug use, incomplete research information, and poor methodological quality in the included studies, the safety of XST needs to be further explored and clarified.

For methodological quality, the risk of bias (ROB) tool was used to facilitate the improved appraisal of evidence. For overall ROB, four studies (10%) were at high ROB due to one domain, and all studies were at low or unclear ROB for one to four domains. For the generation of random sequence, thirteen studies were assessed at low ROB because of the specific methods, including random number table, random in order of visits, coin flipping, the picking method, and SAS12.0 software. Three studies were evaluated at high risk of selection bias, as randomization was achieved by alternating the two treatments, so the intervention allocations could have been foreseen in advance. All included studies were assessed at unclear risk of selection, performance, and detection bias since they did not provide information to permit judgement about high or low ROB associated with the allocation concealment, blinding of participants and personnel, and blinding of outcome assessment. One included study was assessed at high risk of attrition bias since there was one subject who left the study due to an unsatisfactory effect of the initial treatment in the experimental group. The remaining included studies were evaluated at low risk of attrition bias. All included studies were free from the risk of selective reporting for all outcomes and were assessed as low risk. We judged all studies to be free from other sources of bias due to the balance baseline characteristics (age, sex, severity of ACI). The GRADE system was applied to evaluate the evidence of efficacy and safety outcomes. For overall response rate and incidence of adverse reactions, the evidence was deemed low quality due to high ROB (selection, performance, detection, and attrition bias) and potential reporting bias (publication bias). For the NIHSS score, ADL score, and IL-6 levels, the quality of evidence was very low, down-graded three times for serious ROB (selection, performance, detection, and attrition bias), inconsistency (*I* ^2^ > 90% for heterogeneity), and potential reporting bias (publication bias). The hs-CRP levels were graded as low-quality evidence due to high ROB (selection, performance, detection, and attrition bias) and potential reporting bias (publication bias). In the present study, the overall quality of evidence for intervention efficacy and safety was low for all outcomes, due to high ROB, inconsistency, and potential reporting bias. More large-sample and high-quality RCTs are needed to improve the level of evidence for the efficacy and safety of using XST combined with CTs to treat patients with ACI. The recommendations of this meta-analysis should be considered in combination with the following aspects: lack of high-quality evidence, uncertain or different values and preferences, and whether uncertain net benefits are worth the cost.

There are a number of limitations to this study that need to be acknowledged. Firstly, the overall methodological quality of the included studies was predominantly poor, largely due to high ROB (selection, performance, detection, and attrition bias). Secondly, the substantial heterogeneity was identified from the NIHSS score, ADL score, and hs-CRP and IL-6 levels, which affected the stability of the results in these outcomes. Third, the reporting of conventional treatments and adverse events was generally inadequate, and all trials did not mention specific treatment programs and complications of treatment at all. Where adverse events were reported, these often consisted of short statements of the absence of adverse events in the study results or discussion without any indication of systematic recording. Fourth, the overall quality of evidence of this study was low because of the high risk of bias, inconsistency, and potential reporting bias. Finally, most of the included studies did not undergo long-term follow-up, so the long-term efficacy of XST cannot be assessed. In addition, no recurrence, disability, or mortality rates were reported in any of the included studies.

According to the results of this study, the use of XST combined with CTs can be used as a reference treatment scheme for clinicians to treat ACI in the future. In the present study, CTs for ACI are diverse, and include thrombolytic drugs, anticoagulants, antiplatelets, antihypertensives, statins, neuroprotective agents, collateral circulation drugs, and lipid-lowering medications. Some drugs have the effect of dilating cerebral vessels, which may have led to the aggravation of ischemia in the ischemic necrotic areas of ACI and enlargement of the lesion (Tang et al., 2018). Clinicians should always pay attention to changes in patients with ACI and choose drugs reasonably and accurately during CTs to avoid the side effects of drugs.

The quality of evidence, the balance between expected and undesirable effects, value and preference, and cost are the four key factors that affect the strength of recommendations in the GRADE system. According to the above four key factors, the recommended strength in the GRADE system is divided into two levels of “strong” and “weak” (Balshem et al., 2011; Guyatt et al., 2011a). In this study, the overall quality of evidence was graded as low for all included studies. In the absence of high-level, evidence-based results, consistent recommendations can be reached through a strict expert consensus method. The Consensus Development Conference (CDC), Nominal Group Technique (NGT), and Delphi method are three specific expert consensus methods commonly used in the medical field (McMillan et al., 2016). We will use the CDC and Delphi method based on the level of evidence obtained, and an expert consensus will be formed on recommendations to revise TCM clinical practice guidelines of XST combined with CTs for ACI. Clinicians should combine evidence-based results, doctors’ clinical experience, and patients’ personal values when making clinical decisions.

In conclusion, our study showed that XST combined with CTs is conditionally recommended to improve the overall response rate and activities of daily living, remedy neurological deficits, and reduce the level of hs-CRP and IL-6 for patients with ACI. There were no serious adverse events observed in this study. The GRADE assessment indicates that the overall quality of this evidence is low. Further high-quality and large-sample RCTs that meet the CONSORT guideline are needed to improve the level of evidence in this study.

## Data Availability Statement****

The data supporting the findings of this study are available within the article and its **Supplementary Materials**.

## Author Contributions

JL and YX had the idea for the study design. JL retrieved literature, extracted data, analyzed data, and wrote this manuscript. MS and LZ extracted the data. All authors contributed to the article and approved the submitted version.

## Funding

This study was supported by the National Key Research and Development Program of China (2018YFC1707400).

## Conflict of Interest

The authors declare that the research was conducted in the absence of any commercial or financial relationships that could be construed as a potential conflict of interest.
