# Supplementary material for: Efficacy and Safety of Xueshuantong Injection on Acute Cerebral Infarction: Clinical Evidence and GRADE Assessment
Source: Front Pharmacol. 2020 Jul 2;11:822. doi: 10.3389/fphar.2020.00822 (PMC7345308; doi:10.3389/fphar.2020.00822)
Supplement: Supplementary file 1 [file DataSheet_1.docx]

Summary table of all of the papers cited

(Efficacy and safety of Xueshuantong injection on acute cerebral infarction- Clinical evidence and GRADE assessment)

| Study | Source | Species, concentration | Quality control reported?(Y/N) | Chemical analysis reported?(Y/N) |
| --- | --- | --- | --- | --- |
| Sun et al., (2015) | Exp Ther Med. doi:10.3892/etm.2015.2462. | Not applicable | Not applicable | Not applicable |
| Arboix and Alio, 2012 | Curr Cardiol Rev. doi:10.2174/157340312801215791. | Not applicable | Not applicable | Not applicable |
| Mokin et al., 2014 | Neurosurg Clin N Am. doi:10.1016/j.nec.2014.04.013. | Not applicable | Not applicable | Not applicable |
| Hsieh and Chiou, 2014 | J Stroke. doi:10.5853/jos.2014.16.2.59. | Not applicable | Not applicable | Not applicable |
| Atik et al., 2016 | Am J Emerg Med. doi:10.1016/j.ajem.2016.08.062. | Not applicable | Not applicable | Not applicable |
| Kim et al., 2012 | Atherosclerosis. doi:10.1016/j.atherosclerosis.2012.06.05. | Not applicable | Not applicable | Not applicable |
| Yong et al., 2013 | Cardiol Rev. doi: 10.1097/CRD.0b013e3182748d37 | Not applicable | Not applicable | Not applicable |
| Chen et al., 2018 | Exp Ther Med. doi:10.3892/etm.2018.6868. | Not applicable | Not applicable | Not applicable |
| Lee et al., 2017 | Neural Regen Res. doi:10.4103/1673-5374.202915. | Not applicable | Not applicable | Not applicable |
| Asahi et al., 2000 | J Cereb Blood Flow Metab. 20, 452–457. | Not applicable | Not applicable | Not applicable |
| Zhang et al., 2017 | Sci Rep. doi:10.1038/s41598-017-03039-7. | Not applicable | Not applicable | Not applicable |
| Jia et al., 2017 | Molecules. doi:10.3390/molecules22050810. | Not applicable | Not applicable | Not applicable |
| Hu and Xu, 2014 | TrAC-Trends Anal Chem. doi: 10.1016/j.trac.2014.06.007. | Not applicable | Not applicable | Not applicable |
| Xiao et al., 2014 | J Pharm Biomed Anal. doi:10.1016/j.jpba.2013.12.042. | Not applicable | Not applicable | Not applicable |
| Ye et al., 2015 | Oncol. Lett. doi:10.3892/ol.2015.3459. | Not applicable | Not applicable | Not applicable |
| Zhong et al., 2015 | Am J Kidney Dis. doi:10.1053/j.ajkd.2015.04.013. | Not applicable | Not applicable | Not applicable |
| Li et al., 2018 | Guangxi Wuzhou Pharmaceutical (Group) Co., Ltd. | Panax notoginseng (Burk.) F. H. Chen, 35mg/ml | Y - Prepared according to People's Republic of China Pharmacopoeia | N |
| Wang et al., 2015 | Guangxi Wuzhou Pharmaceutical (Group) Co., Ltd. | Panax notoginseng (Burk.) F. H. Chen, 35mg/ml | Y - Prepared according to People's Republic of China Pharmacopoeia | N |
| Huang et al., 2013 | Guangxi Wuzhou Pharmaceutical (Group) Co., Ltd. | Panax notoginseng (Burk.) F. H. Chen, 35mg/ml | Y - Prepared according to People's Republic of China Pharmacopoeia | N |
| Zhao et al., 2013 | Guangxi Wuzhou Pharmaceutical (Group) Co., Ltd. | Panax notoginseng (Burk.) F. H. Chen, 35mg/ml | Y - Prepared according to People's Republic of China Pharmacopoeia | N |
| Li et al., 2009 | Guangxi Wuzhou Pharmaceutical (Group) Co., Ltd. | Panax notoginseng (Burk.) F. H. Chen, 35mg/ml | Y - Prepared according to People's Republic of China Pharmacopoeia | N |
| Lei et al., 2007 | Guangxi Wuzhou Pharmaceutical (Group) Co., Ltd. | Panax notoginseng (Burk.) F. H. Chen, 35mg/ml | Y - Prepared according to People's Republic of China Pharmacopoeia | N |
| Dong et al., 2003 | Chinese Herbal Medicine in Yunnan Province, China | Panax notoginseng (Burk.) F. H. Chen, 35mg/ml | Y - Prepared according to People's Republic of China Pharmacopoeia | Y - HPLC |
| Shen et al., 2017 | Chinese Herbal Medicine in Yunnan Province, China | Panax notoginseng (Burk.) F. H. Chen, 35mg/ml | Y - Prepared according to People's Republic of China Pharmacopoeia | Y - UPLC BEH C18 |
| Zhong et al., 2005 | Chinese Herbal Medicine in Yunnan Province, China | Panax notoginseng (Burk.) F. H. Chen, 35mg/ml | Y - Prepared according to People's Republic of China Pharmacopoeia | N |
| Wang et al., 2013 | Chinese Herbal Medicine in Yunnan Province, China | Panax notoginseng (Burk.) F. H. Chen, 35mg/ml | Y - Prepared according to People's Republic of China Pharmacopoeia | N |
| Toh et al., 2010 | Chinese Herbal Medicine in Yunnan Province, China | Panax notoginseng (Burk.) F. H. Chen, 35mg/ml | Y - Prepared according to People's Republic of China Pharmacopoeia | Y - UHPLC/TOFMS |
| Wang et al., 2012 | Chinese Herbal Medicine in Yunnan Province, China | Panax notoginseng (Burk.) F. H. Chen, 35mg/ml | Y - Prepared according to People's Republic of China Pharmacopoeia | Y - HPLC |
| Li et al., 2012 | Chinese Herbal Medicine in Yunnan Province, China | Panax notoginseng (Burk.) F. H. Chen, 35mg/ml | Y - Prepared according to People's Republic of China Pharmacopoeia | N |
| Ye and Yang, et al., 2011 | Chinese Herbal Medicine in Yunnan Province, China | Panax notoginseng (Burk.) F. H. Chen, 35mg/ml | Y - Prepared according to People's Republic of China Pharmacopoeia | N |
| Ye and Kong, et al., 2011 | Chinese Herbal Medicine in Yunnan Province, China | Panax notoginseng (Burk.) F. H. Chen, 35mg/ml | Y - Prepared according to People's Republic of China Pharmacopoeia | N |
| Ye and Zhang, et al., 2011 | Chinese Herbal Medicine in Yunnan Province, China | Panax notoginseng (Burk.) F. H. Chen, 35mg/ml | Y - Prepared according to People's Republic of China Pharmacopoeia | N |
| Ye et al., 2009 | Chinese Herbal Medicine in Yunnan Province, China | Panax notoginseng (Burk.) F. H. Chen, 35mg/ml | Y - Prepared according to People's Republic of China Pharmacopoeia | N |
| Li et al., 2010 | Chinese Herbal Medicine in Yunnan Province, China | Panax notoginseng (Burk.) F. H. Chen, 35mg/ml | Y - Prepared according to People's Republic of China Pharmacopoeia | N |
| Zhang et al., 2013 | Chinese Herbal Medicine in Yunnan Province, China | Panax notoginseng (Burk.) F. H. Chen, 35mg/ml | Y - Prepared according to People's Republic of China Pharmacopoeia | N |
| Hu et al., 2013 | Chinese Herbal Medicine in Yunnan Province, China | Panax notoginseng (Burk.) F. H. Chen, 35mg/ml | Y - Prepared according to People's Republic of China Pharmacopoeia | N |
| Zhang et al., 2014 | Chinese Herbal Medicine in Yunnan Province, China | Panax notoginseng (Burk.) F. H. Chen, 35mg/ml | Y - Prepared according to People's Republic of China Pharmacopoeia | N |
| Liberati et al., 2009 | *BMJ*. doi:10.1136/bmj.b2700. | Not applicable | Not applicable | Not applicable |
| Cochrane Consumers and Communication, 2016 | Cochrane Consumers and Communication | Not applicable | Not applicable | Not applicable |
| Higgins et al., 2011 | *BMJ*. doi: 10.1136/bmj.d5928. | Not applicable | Not applicable | Not applicable |
| Higgins and Thompson, 2002 | *Stat Med.* 21, 1539–1558. | Not applicable | Not applicable | Not applicable |
| Higgins et al., 2003 | *BMJ.* 327, 557–560. | Not applicable | Not applicable | Not applicable |
| Guyatt et al., 2011 | *J Clin Epidemiol*. 64, 383–394. | Not applicable | Not applicable | Not applicable |
| Balshem et al., 2011 | *J Clin Epidemiol*. 64, 401–406. | Not applicable | Not applicable | Not applicable |
| Zhang A et al., 2019 | Guangdong Lei Yun Shang Pharmaceutical Co., Ltd. | Panax notoginseng (Burk.) F. H. Chen, 35mg/ml | Y - Prepared according to People's Republic of China Pharmacopoeia | N |
| Zhang B et al., 2019 | Guangxi Wuzhou Pharmaceutical (Group) Co., Ltd. | Panax notoginseng (Burk.) F. H. Chen, 35mg/ml | Y - Prepared according to People's Republic of China Pharmacopoeia | N |
| Xu et al., 2019 | Guangxi Wuzhou Pharmaceutical (Group) Co., Ltd. | Panax notoginseng (Burk.) F. H. Chen, 35mg/ml | Y - Prepared according to People's Republic of China Pharmacopoeia | N |
| Liu and Wang, 2019 | Not mentioned | Panax notoginseng (Burk.) F. H. Chen, 35mg/ml | Y - Prepared according to People's Republic of China Pharmacopoeia | N |
| Li, 2019 | Lizhu pharmaceutical group co., Ltd. | Panax notoginseng (Burk.) F. H. Chen, 35mg/ml | Y - Prepared according to People's Republic of China Pharmacopoeia | N |
| Du, 2019 | Harbin Shengtai Biopharmaceutical Co., Ltd. | Panax notoginseng (Burk.) F. H. Chen, 35mg/ml | Y - Prepared according to People's Republic of China Pharmacopoeia | N |
| Wang et al., 2018 | Guangxi Wuzhou Pharmaceutical (Group) Co., Ltd. | Panax notoginseng (Burk.) F. H. Chen, 35mg/ml | Y - Prepared according to People's Republic of China Pharmacopoeia | N |
| Ren et al., 2018 | Guangxi Wuzhou Pharmaceutical (Group) Co., Ltd. | Panax notoginseng (Burk.) F. H. Chen, 35mg/ml | Y - Prepared according to People's Republic of China Pharmacopoeia | N |
| Chen, 2018 | Not mentioned | Panax notoginseng (Burk.) F. H. Chen, 35mg/ml | Y - Prepared according to People's Republic of China Pharmacopoeia | N |
| Chen and Ding, 2018 | Guangdong Yongkang Pharmaceutical Co., Ltd. | Panax notoginseng (Burk.) F. H. Chen, 35mg/ml | Y - Prepared according to People's Republic of China Pharmacopoeia | N |
| Shu, 2018 | Not mentioned | Panax notoginseng (Burk.) F. H. Chen, 35mg/ml | Y - Prepared according to People's Republic of China Pharmacopoeia | N |
| Gu and Yang, 2017 | Lizhu pharmaceutical group co., Ltd. | Panax notoginseng (Burk.) F. H. Chen, 35mg/ml | Y - Prepared according to People's Republic of China Pharmacopoeia | N |
| Zhang et al., 2016 | Guangxi Wuzhou Pharmaceutical (Group) Co., Ltd. | Panax notoginseng (Burk.) F. H. Chen, 35mg/ml | Y - Prepared according to People's Republic of China Pharmacopoeia | N |
| Jiao et al., 2016 | Lizhu pharmaceutical group co., Ltd. | Panax notoginseng (Burk.) F. H. Chen, 35mg/ml | Y - Prepared according to People's Republic of China Pharmacopoeia | N |
| Zheng, 2016 | Lizhu pharmaceutical group co., Ltd. | Panax notoginseng (Burk.) F. H. Chen, 35mg/ml | Y - Prepared according to People's Republic of China Pharmacopoeia | N |
| Yang and Wei, 2015 | Guangdong Lei Yun Shang Pharmaceutical Co., Ltd. | Panax notoginseng (Burk.) F. H. Chen, 35mg/ml | Y - Prepared according to People's Republic of China Pharmacopoeia | N |
| Liu, 2015 | Guangxi Wuzhou Pharmaceutical (Group) Co., Ltd. | Panax notoginseng (Burk.) F. H. Chen, 35mg/ml | Y - Prepared according to People's Republic of China Pharmacopoeia | N |
| Li, 2015 | Not mentioned | Panax notoginseng (Burk.) F. H. Chen, 35mg/ml | Y - Prepared according to People's Republic of China Pharmacopoeia | N |
| He et al., 2015 | Guangxi Wuzhou Pharmaceutical (Group) Co., Ltd. | Panax notoginseng (Burk.) F. H. Chen, 35mg/ml | Y - Prepared according to People's Republic of China Pharmacopoeia | N |
| Chen, 2015 | Guangxi Wuzhou Pharmaceutical (Group) Co., Ltd. | Panax notoginseng (Burk.) F. H. Chen, 35mg/ml | Y - Prepared according to People's Republic of China Pharmacopoeia | N |
| Zhang, 2014 | Guangxi Wuzhou Pharmaceutical (Group) Co., Ltd. | Panax notoginseng (Burk.) F. H. Chen, 35mg/ml | Y - Prepared according to People's Republic of China Pharmacopoeia | N |
| Wang, 2014 | Not mentioned | Panax notoginseng (Burk.) F. H. Chen, 35mg/ml | Y - Prepared according to People's Republic of China Pharmacopoeia | N |
| Li, 2014 | Not mentioned | Panax notoginseng (Burk.) F. H. Chen, 35mg/ml | Y - Prepared according to People's Republic of China Pharmacopoeia | N |
| Zhang, 2013 | Guangxi Wuzhou Pharmaceutical (Group) Co., Ltd. | Panax notoginseng (Burk.) F. H. Chen, 35mg/ml | Y - Prepared according to People's Republic of China Pharmacopoeia | N |
| Wang, 2013 | Guangxi Wuzhou Pharmaceutical (Group) Co., Ltd. | Panax notoginseng (Burk.) F. H. Chen, 35mg/ml | Y - Prepared according to People's Republic of China Pharmacopoeia | N |
| Gong and Gong, 2013 | Not mentioned | Panax notoginseng (Burk.) F. H. Chen, 35mg/ml | Y - Prepared according to People's Republic of China Pharmacopoeia | N |
| Zheng, 2012 | Not mentioned | Panax notoginseng (Burk.) F. H. Chen, 35mg/ml | Y - Prepared according to People's Republic of China Pharmacopoeia | N |
| Liang, 2012 | Guangxi Wuzhou Pharmaceutical (Group) Co., Ltd. | Panax notoginseng (Burk.) F. H. Chen, 35mg/ml | Y - Prepared according to People's Republic of China Pharmacopoeia | N |
| Li A, 2012 | Not mentioned | Panax notoginseng (Burk.) F. H. Chen, 35mg/ml | Y - Prepared according to People's Republic of China Pharmacopoeia | N |
| Li B, 2012 | Guangxi Wuzhou Pharmaceutical (Group) Co., Ltd. | Panax notoginseng (Burk.) F. H. Chen, 35mg/ml | Y - Prepared according to People's Republic of China Pharmacopoeia | N |
| Duan and Shang, 2012 | Not mentioned | Panax notoginseng (Burk.) F. H. Chen, 35mg/ml | Y - Prepared according to People's Republic of China Pharmacopoeia | N |
| Wang, 2011 | Not mentioned | Panax notoginseng (Burk.) F. H. Chen, 35mg/ml | Y - Prepared according to People's Republic of China Pharmacopoeia | N |
| Sun, 2011 | Guangxi Wuzhou Pharmaceutical (Group) Co., Ltd. | Panax notoginseng (Burk.) F. H. Chen, 35mg/ml | Y - Prepared according to People's Republic of China Pharmacopoeia | N |
| Li, 2011 | Not mentioned | Panax notoginseng (Burk.) F. H. Chen, 35mg/ml | Y - Prepared according to People's Republic of China Pharmacopoeia | N |
| Gao, 2010 | Not mentioned | Panax notoginseng (Burk.) F. H. Chen, 35mg/ml | Y - Prepared according to People's Republic of China Pharmacopoeia | N |
| Zhao, 2010 | Not mentioned | Panax notoginseng (Burk.) F. H. Chen, 35mg/ml | Y - Prepared according to People's Republic of China Pharmacopoeia | N |
| Ma, 2010 | Not mentioned | Panax notoginseng (Burk.) F. H. Chen, 35mg/ml | Y - Prepared according to People's Republic of China Pharmacopoeia | N |
| Wang, 2009 | Guangxi Wuzhou Pharmaceutical (Group) Co., Ltd. | Panax notoginseng (Burk.) F. H. Chen, 35mg/ml | Y - Prepared according to People's Republic of China Pharmacopoeia | N |
| Wang et al., 2009 | Guangxi Wuzhou Pharmaceutical (Group) Co., Ltd. | Panax notoginseng (Burk.) F. H. Chen, 35mg/ml | Y - Prepared according to People's Republic of China Pharmacopoeia | N |
| Yang et al., 2004 | Lizhu pharmaceutical group co., Ltd. | Panax notoginseng (Burk.) F. H. Chen, 35mg/ml | Y - Prepared according to People's Republic of China Pharmacopoeia | N |
| FDA, 2007 | http://www.fda.gov/downloads/drugs/guidancecomplianceregulatoryinformation/guidances/ucm071590.pdf. | Not applicable | Not applicable | Not applicable |
| Wu et al., 2019 | Postgrad Med J. doi:10.1136/postgradmedj-2019-136398. | Not applicable | Not applicable | Not applicable |
| Chen et al., 2018 | Exp Ther Med. doi:10.3892/etm.2018.6868. | Not applicable | Not applicable | Not applicable |
| Segers et al., 2007 | Circulation. doi:10.1161/CIRCULATIONAHA.106.636415. | Not applicable | Not applicable | Not applicable |
| Moreno et al., 2004 | Circulation. doi:10.1161/01.CIR.0000143233.87854.23. | Not applicable | Not applicable | Not applicable |
| Tang et al., 2018 | Chinese Journal of Clinical Rational Drug Use. 11, 179-181. | Not applicable | Not applicable | Not applicable |
| Guyatt et al., 2011 | J Clin Epidemiol. 64, 1311-6. | Not applicable | Not applicable | Not applicable |
| Moynihan et al., 2016 | Oslo: Norwegian Knowledge Centre for the Health Services | Not applicable | Not applicable | Not applicable |

| **Four main components of Panax notoginseng (Burk.) F. H. Chen in Xueshuantong injection**  **(**Guangxi Wuzhou Pharmaceutical (Group) Co., Ltd.**)** | | | |
| --- | --- | --- | --- |
| Name | Molecular Formula | Proportion | Functions |
| notoginsenoside R1 | C_47_H_80_O_18_ | ≥8.5% | It has a role as a plant metabolite, an antioxidant, a neuroprotective agent, an apoptosis inducer and a phytoestrogen. |
| ginsenoside Rg1 | C_42_H_72_O_14_ | ≥30% | It has a role as a neuroprotective agent and a pro-angiogenic agent. |
| ginsenoside Rd | C_48_H_82_O_18_ | ≥1.4% | It has a role as a vulnerary, a neuroprotective agent, an apoptosis inducer, an anti-inflammatory drug, an immunosuppressive agent and a plant metabolite. |
| ginsenoside Rb1 | C_54_H_92_O_23_ | ≥15.5% | It has a role as a neuroprotective agent, an anti-obesity agent, an anti-inflammatory drug, an apoptosis inhibitor, a radical scavenger and a plant metabolite. |
