# Supplementary figures and images for: Efficacy and Safety of Xueshuantong Injection on Acute Cerebral Infarction: Clinical Evidence and GRADE Assessment
Source: Front Pharmacol. 2020 Jul 2;11:822. doi: 10.3389/fphar.2020.00822 (PMC7345308; doi:10.3389/fphar.2020.00822)

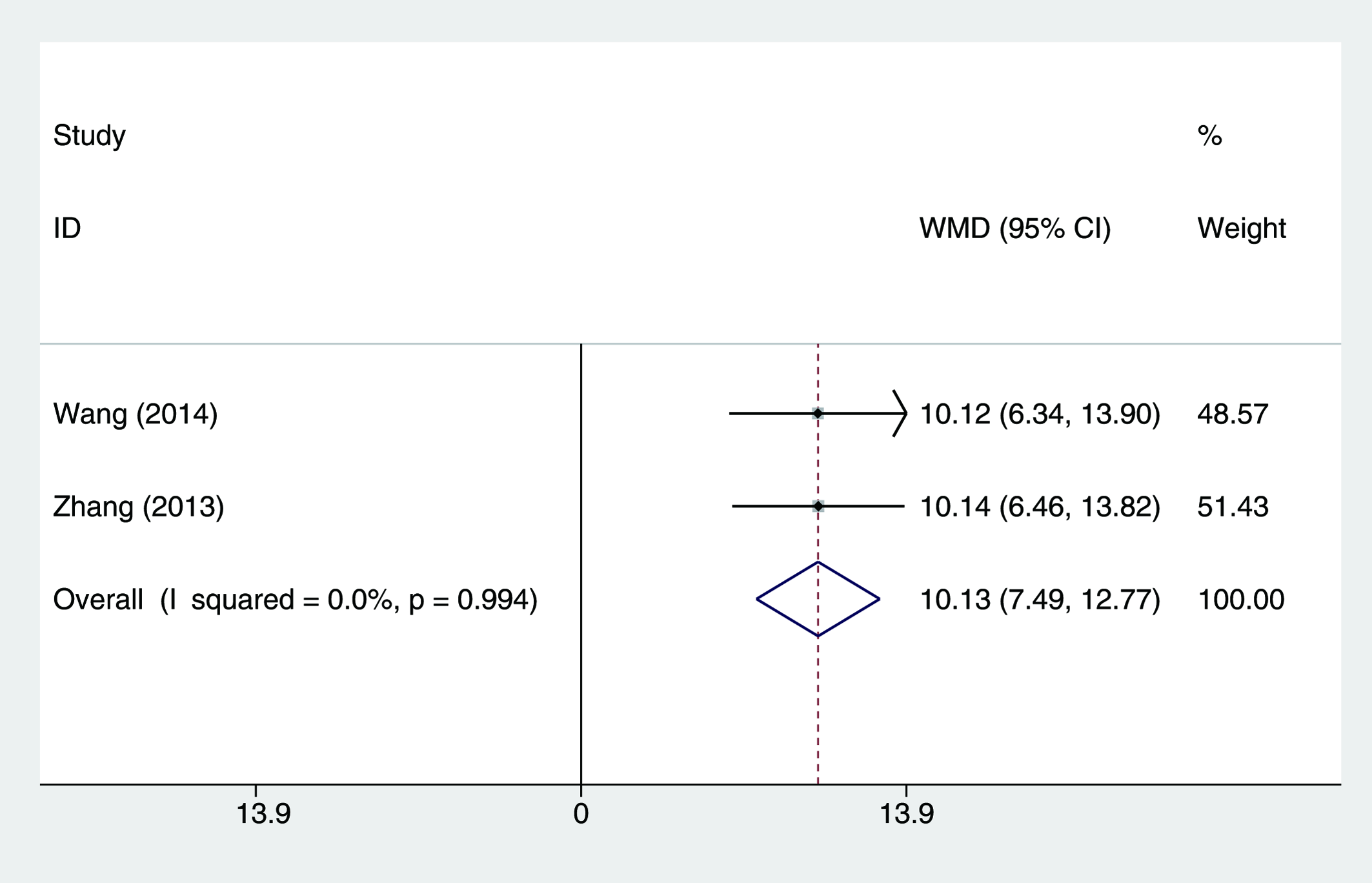

Supplement: Supplementary file 3 [file Image_1.tif]

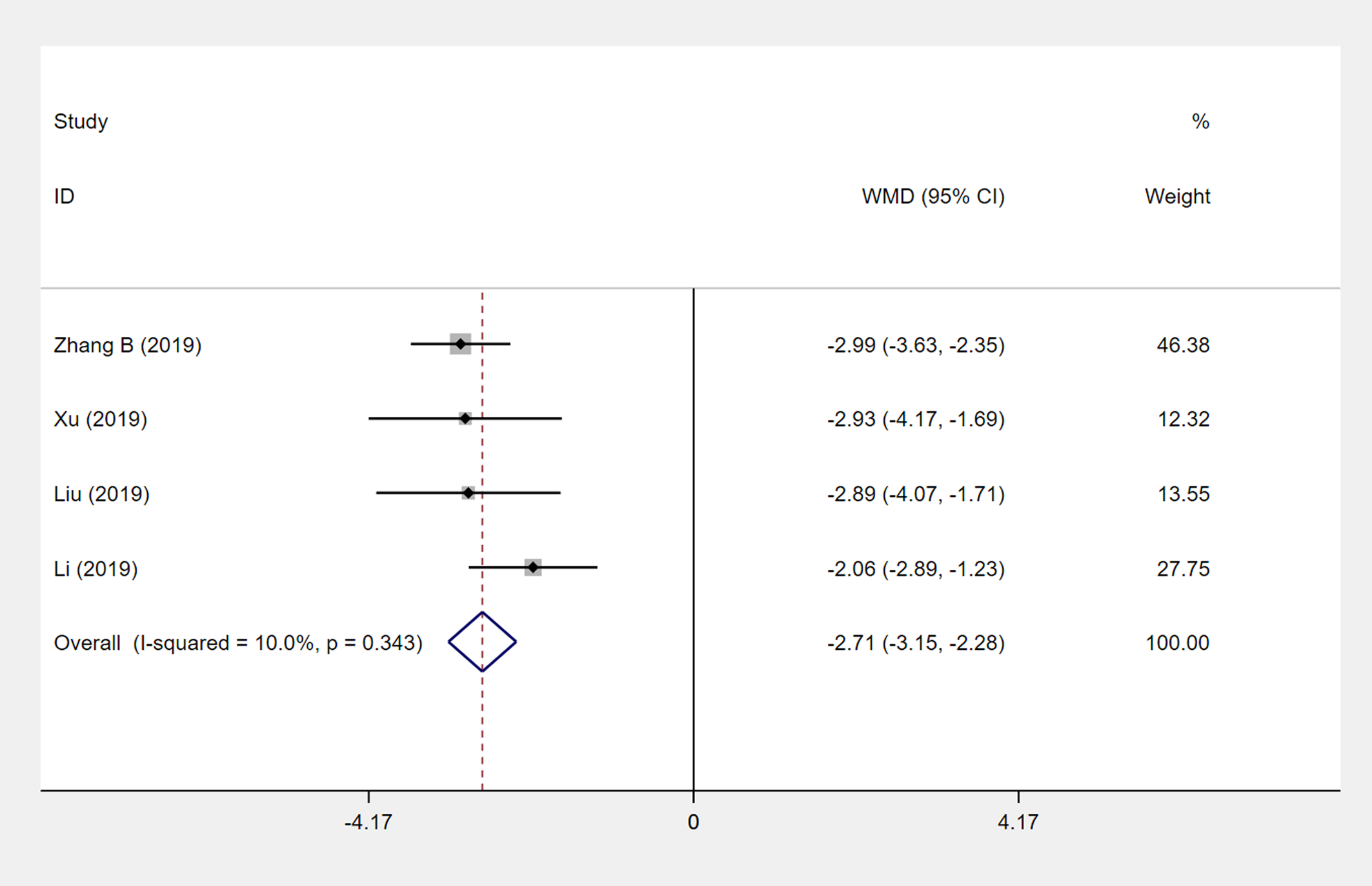

Supplement: Supplementary file 4 [file Image_2.tif]

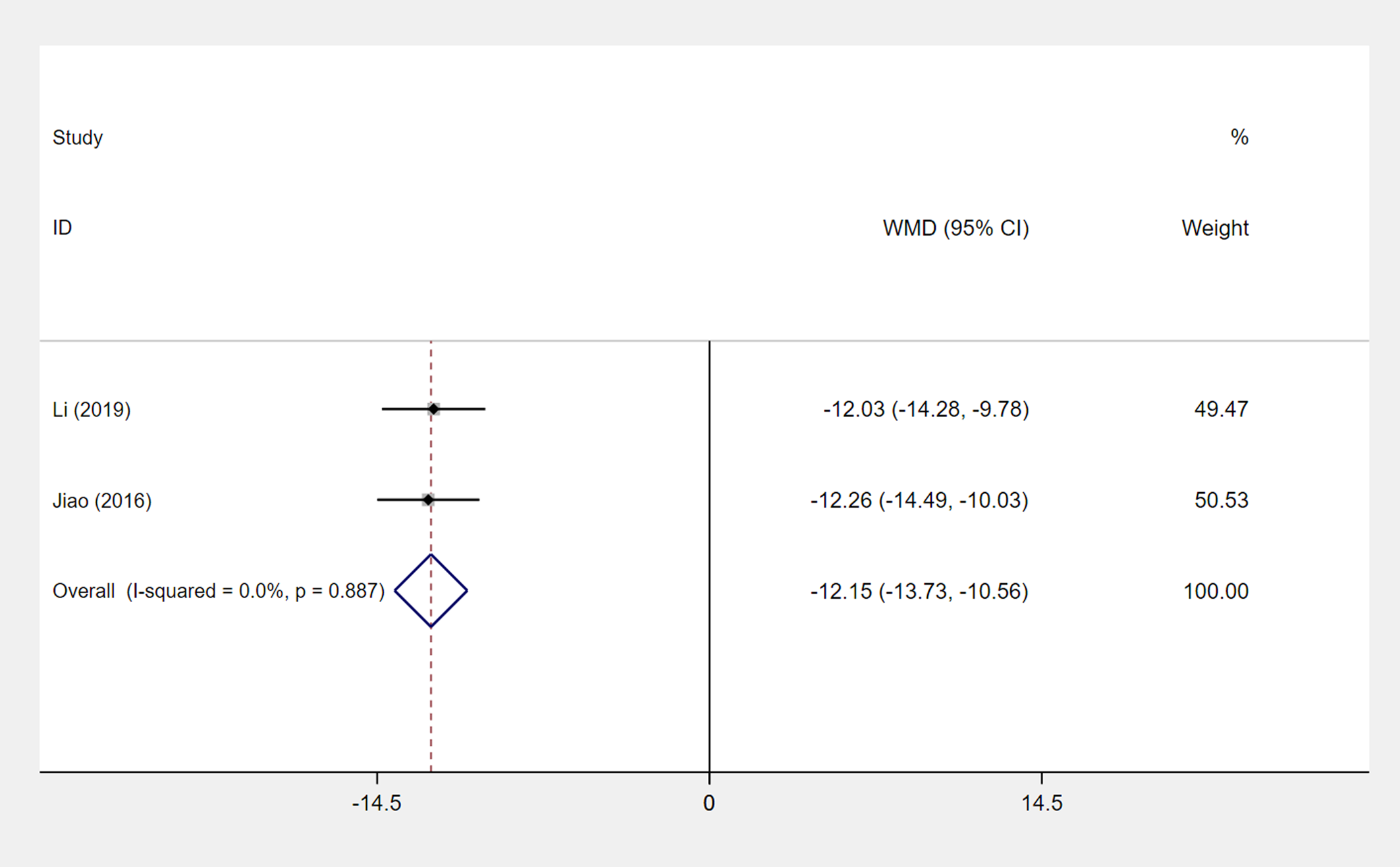

Supplement: Supplementary file 5 [file Image_3.tif]

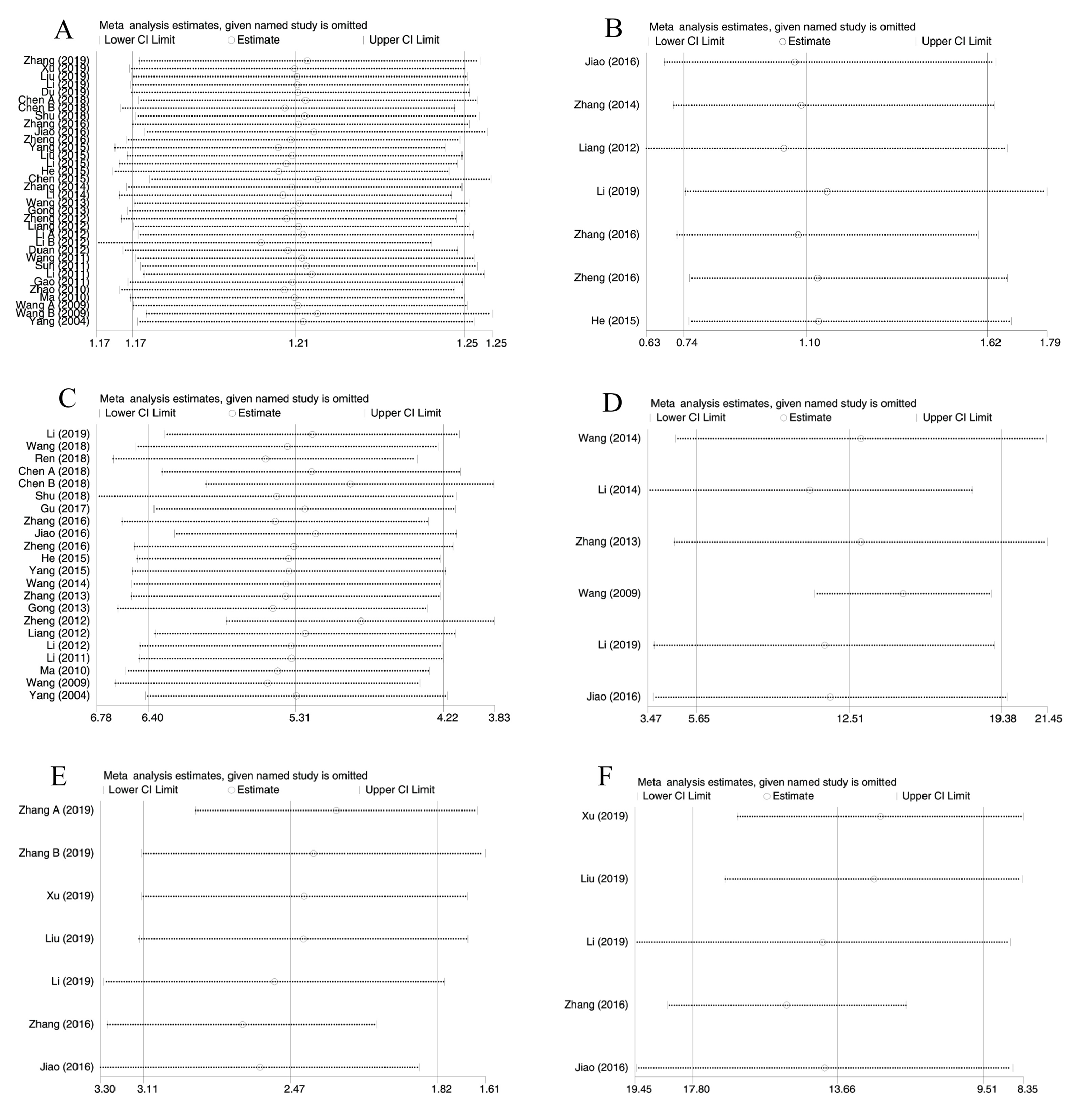

Supplement: Supplementary file 6 [file Image_4.tif]
