# Supplementary material for: Efficacy and Safety of Xueshuantong Injection on Acute Cerebral Infarction: Clinical Evidence and GRADE Assessment
Source: Front Pharmacol. 2020 Jul 2;11:822. doi: 10.3389/fphar.2020.00822 (PMC7345308; doi:10.3389/fphar.2020.00822)
Supplement: Supplementary file 7 [file Table_1.docx]

| Table S1 Four main components of PNS in XST | | | | |
| --- | --- | --- | --- | --- |
| Name | Molecular Formula | Molecular Weight | Functions | Source (ChEBI database) |
| notoginsenoside R1 | C_47_H_80_O_18_ | 933.1 g/mol | It has a role as a plant metabolite, an antioxidant, a neuroprotective agent, an apoptosis inducer and a phytoestrogen. | URL:http://www.ebi.ac.uk/chebi/searchId.do?chebiId=CHEBI:77149 |
| ginsenoside Rg1 | C_42_H_72_O_14_ | 801 g/mol | It has a role as a neuroprotective agent and a pro-angiogenic agent. | URL: <http://www.ebi.ac.uk/chebi/searchId.do?chebiId=CHEBI:67987> |
| ginsenoside Rd | C_48_H_82_O_18_ | 947.2 g/mol | It has a role as a vulnerary, a neuroprotective agent, an apoptosis inducer, an anti-inflammatory drug, an immunosuppressive agent and a plant metabolite. | URL:http://www.ebi.ac.uk/chebi/searchId.do?chebiId=CHEBI:67988 |
| ginsenoside Rb1 | C_54_H_92_O_23_ | 1109.3 g/mol | It has a role as a neuroprotective agent, an anti-obesity agent, an anti-inflammatory drug, an apoptosis inhibitor, a radical scavenger and a plant metabolite. | URL:http://www.ebi.ac.uk/chebi/searchId.do?chebiId=CHEBI:67989 |
| Description: Chemical Entities of Biological Interest (ChEBI) is a database and ontology of molecular entities focused on 'small' chemical compounds, that is part of the Open Biomedical Ontologies effort. The term "molecular entity" refers to any constitutionally or isotopically distinct atom, molecule, ion, ion pair, radical, radical ion, complex, conformer, etc., identifiable as a separately distinguishable entity. PNS: Panax notoginseng saponins. XST: Xueshuantong injection. | | | | |
