# Supplementary material for: Efficacy and Safety of Xueshuantong Injection on Acute Cerebral Infarction: Clinical Evidence and GRADE Assessment
Source: Front Pharmacol. 2020 Jul 2;11:822. doi: 10.3389/fphar.2020.00822 (PMC7345308; doi:10.3389/fphar.2020.00822)
Supplement: Supplementary file 8 [file Table_2.docx]

| **Table S2. The search strategy for PubMed** | |
| --- | --- |
| **Number** | **Search terms** |
| #1 | (acute ischemic stroke[MeSH Terms]) OR acute ischemic stroke |
| #2 | ((XST[Title/Abstract]) OR Xueshuantong injection [Title/Abstract]) OR Xueshuantong [Title/Abstract] |
| #3 | (RCT[Title/Abstract]) OR randomized controlled trial[Title/Abstract] |
| #4 | (Efficacy[Title/Abstract]) OR Safety[Title/Abstract] |
| #5 | #1 and #2 and #3 and #4 |
