# Supplementary material for: Efficacy and Safety of Xueshuantong Injection on Acute Cerebral Infarction: Clinical Evidence and GRADE Assessment
Source: Front Pharmacol. 2020 Jul 2;11:822. doi: 10.3389/fphar.2020.00822 (PMC7345308; doi:10.3389/fphar.2020.00822)
Supplement: Supplementary file 9 [file Table_3.docx]

| **Table S3 Study and participant characteristics** | | | | | | | | | | | |
| --- | --- | --- | --- | --- | --- | --- | --- | --- | --- | --- | --- |
| **Study** | **Reference** | **Sample size** | | | **Gender**  **(T / C)** | **Age / (year)**  **(T / C)** | **Treatment group** | | **Control group** | **Duration / day** | **Outcomes** |
|  |  | **T** | **C** | **Total** |  |  | **Dose of XST**  **mg/ml**  **(injection route)** | **Combined with treatment** |  |  |  |
| Zhang A 2019 | Zhang A et al., 2019 | 109 | 109 | 218 | T: 59/50  C: 47/62 | T: 48～68(61.53±8.49)  C: 46～70(66.20±8.37) | 210mg | CTs | CTs | 15 | ⑤ |
| Zhang B 2019 | Zhang B et al., 2019 | 50 | 50 | 100 | T: 34/16  C: 33/17 | T: 50～80(62.05±3.07)  C: 51～80(61.26±3.04) | 600mg | CTs | CTs | 14 | ①⑤ |
| Xu 2019 | Xu et al., 2019 | 35 | 35 | 70 | T: 25/10  C: 24/11 | T: 69.1±7.7  C: 68.7±7.1 | 300mg | CTs | CTs | 14 | ①⑤⑥ |
| Liu 2019 | Liu and Wang, 2019 | 38 | 38 | 76 | T: 26/12  C: 24/14 | T: 61.7±6.1  C: 62.4±6.3 | 400mg | CTs | CTs | 14 | ①⑤⑥ |
| Li 2019 | Li, 2019 | 50 | 50 | 100 | T: 26/24  C: 27/23 | T: 55～69(61.2±6.9)  C: 56～69(61.7±6.6) | 400mg | CTs | CTs | 14 | ①③④⑤⑥⑦ |
| Du 2019 | Du, 2019 | 53 | 53 | 106 | T: 30/23  C: 29/24 | T: 42～74(57.08±5.38)  C: 41～72(56.76±5.43) | 500mg | CTs | CTs | 14 | ① |
| Wang 2018 | Wang et al., 2018 | 40 | 40 | 80 | T: 21/19  C: 23/17 | T: 49～76(57.3±8.5)  C: 50～74(56.7±9.4) | 450mg | CTs | CTs | 14 | ③ |
| Ren 2018 | Ren et al., 2018 | 40 | 40 | 80 | T: 31/9  C: 33/7 | T: 56～77(64.77±6.98)  C: 55～78(65.95±7.53) | 450mg | CTs | CTs | 14 | ③ |
| Chen A 2018 | Chen, 2018 | 50 | 50 | 100 | T: 28/22  C: 27/23 | T: 53～72(60.3±3.2)  C: 52～72(59.5±3.7) | 100mg | CTs | CTs | 14 | ①③ |
| Chen B 2018 | Chen and Ding, 2018 | 40 | 40 | 80 | T: 21/29  C: 25/15 | T: 58.91±12.91  C: 57.84±13.35 | 400mg | CTs | CTs | 28 | ①③ |
| Shu 2018 | Shu, 2018 | 100 | 100 | 200 | T: 67/33  C: 65/35 | T: 51～83(72.34±12.13)  C: 51～82(72.13±12.56) | 500mg | CTs | CTs | 14 | ①③ |
| Gu  2017 | Gu and Yang, 2017 | 34 | 34 | 68 | 35/33 | (58.8±5.9) | 500mg | CTs | CTs | 14 | ③ |
| Zhang 2016 | Zhang et al., 2016 | 48 | 48 | 96 | T: 26/22  C: 27/21 | T: 50～74(67.38±3.17)  C: 51～76(68.52±3.64) | 300mg | CTs | CTs | 14 | ①③⑤⑥⑦ |
| Jiao 2016 | Jiao et al., 2016 | 68 | 67 | 96 | T: 40/28  C: 39/28 | T: 55～68(63.24±7.6)  C: 57～68(62.4±7.8) | 400mg | CTs | CTs | 14 | ①②③④⑤⑥⑦ |
| Zheng 2016 | Zheng, 2016 | 30 | 30 | 60 | T: 16/14  C: 15/15 | T: 55～74(60.50±1.5)  C: 54～74(61.5±0.5) | 200-500mg | CTs | CTs | 30 | ①③⑦ |
| Yang 2015 | Yang and Wei, 2015 | 42 | 42 | 84 | T: 25/17  C: 28/14 | T: 62～84(71.4±4.9)  C: 60～80(70.4±5.1) | 300mg | CTs | CTs | 14 | ①③⑦ |
| Liu 2015 | Liu, 2015 | 40 | 40 | 80 | T: 25/15  C: 28/12 | T: 62～80  C: 60～80 | 500mg | CTs | CTs | 28 | ①⑦ |
| Li 2015 | Li, 2015 | 66 | 66 | 132 | T: 40/26  C: 35/31 | T: 53.9～87.3(74.1±3.1)  C: 52.3～88.6(73.5±3.6) | 450mg | CTs | CTs | 14 | ①⑦ |
| He 2015 | He et al., 2015 | 63 | 63 | 126 | T: 36/27  C: 38/25 | T: 64.58 ± 7.34  C: 64.92 ± 7.85 | 500mg | CTs | CTs | 28 | ①③⑦ |
| Chen 2015 | Chen, 2015 | 39 | 39 | 78 | T: 25/14  C: 20/19 | T: 48～82(62±1.2)  C: 47～84(63±2.1) | 480mg | CTs | CTs | 18 | ①③ |
| Zhang 2014 | Zhang, 2014 | 44 | 44 | 88 | T: 25/19  C: 28/16 | T: 43～72(54.30±7.90)  C: 42～74(52.90±8.10) | 250mg | CTs | CTs | 14 | ①②⑦ |
| Wang  2014 | Wang, 2014 | 40 | 40 | 80 | 50/30 | 45～78( 56 ± 3.30) | 450mg | CTs | CTs | 15 | ③④ |
| Li 2014 | Li, 2014 | 22 | 20 | 42 | 26/16 | 56～72(65.5±8.2) | 40mg | CTs | CTs | UK | ①③④ |
| Zhang 2013 | Zhang, 2013 | 43 | 42 | 85 | 52/33 | 48～76(55.6±4.6) | 450mg | CTs | CTs | 14 | ③④⑦ |
| Wang 2013 | Wang, 2013 | 25 | 25 | 50 | T: 13/12  C: 13/12 | T: 46～89( 69.88±12.10)  C: 41～89(70.28±11.04) | 400mg | CTs | CTs | 14 | ① |
| Gong 2013 | Gong and Gong, 2013 | 48 | 45 | 93 | T: 25/23  C: 26/19 | T: 36～85  C: 40～83 | 450mg | CTs | CTs | 14 | ①③⑦ |
| Zheng 2012 | Zheng, 2012 | 42 | 42 | 84 | T: 22/20  C: 23/19 | T: 55～76(63.60±6.40)  C: 54～75(62.80±5.80) | 450mg | CTs | CTs | 28 | ①③ |
| Liang 2012 | Liang, 2012 | 50 | 50 | 100 | T: 31/19  C: 28/12 | T: 62.9  C: 66.5 | 300mg | CTs | CTs | 14 | ①②③⑦ |
| Li A 2012 | Li A, 2012 | 30 | 30 | 60 | T: 17/13  C: 16/14 | T: 55～70  C: 57～68 | 450mg | CTs | CTs | 14 | ①③ |
| Li B  2012 | Li B, 2012 | 100 | 100 | 200 | 117/83 | 65.6(38～87) | 300-450mg | CTs | CTs | 14 | ① |
| Duan 2012 | Duan and Shang, 2012 | 38 | 38 | 76 | T: 24/14  C: 22/16 | T: 57.5(35～80)  C: 64.5(48～79) | 450mg | CTs | CTs | 15 | ①⑦ |
| Wang  2011 | Wang, 2011 | 50 | 40 | 90 | T: 30/20  C: 22/18 | T:70(50～80)  C:72(55～85) | 500mg | CTs | CTs | 14 | ①⑦ |
| Sun 2011 | Sun, 2011 | 36 | 34 | 70 | T: 20/16  C: 19/15 | T: 63.5( 51～26)  C: 62.9( 54～24) | 300-450mg | CTs | CTs | 15 | ① |
| Li 2011 | Li, 2011 | 45 | 45 | 90 | 48/32 | 52～78( 62.4 ± 5.2) | 250mg | CTs | CTs | 14 | ①③⑦ |
| Gao 2010 | Gao, 2010 | 48 | 48 | 96 | 52/44 | 62(46～74) | 400mg | CTs | CTs | 14 | ①⑦ |
| Zhao 2010 | Zhao, 2010 | 50 | 48 | 98 | T: 29/21  C: 29/19 | T: 68.5(52～81)  C: 66.7(53～80) | 700mg | CTs | CTs | 14 | ① |
| Ma  2010 | Ma, 2010 | 32 | 28 | 60 | T: 18/14  C: 16/12 | T: 61.5(48～75)  C: 64(50～78) | 450mg | CTs | CTs | UK | ①③⑦ |
| Wang A 2009 | Wang, 2009 | 30 | 30 | 60 | T: 19/11  C: 17/13 | T: 61.0±8.99  C: 63.5±9.48 | 450mg | CTs | CTs | 14 | ①③④⑦ |
| Wang B 2009 | Wang et al., 2009 | 110 | 100 | 210 | T: 59/51  C: 59/41 | UK | 300mg | CTs | CTs | 14 | ① |
| Yang 2004 | Yang et al., 2004 | 76 | 30 | 106 | T: 49/27  C: 19/11 | T: 48～74(62.5 ±15.4 )  C: 47～75(62.3 ±16.5) | 350mg | CTs | CTs | 15 | ①③ |
| T: Treatment group; C: Control group; XST: Xueshuantong injection; CTs: Conventional treatments: thrombolytic drugs, anticoagulant, antiplatelet, antihypertensive, statins, neuroprotective agents, collateral circulation drugs and lipid-lowering medications; UK: Unknown; ①: Overall response rate; ②: Incidence of adverse reactions; ③: NIHSS score; ④: Activities of daily living score; ⑤: High sensitivity C-reactive protein; ⑥: Interleukin-6; ⑦: Adverse events. | | | | | | | | | | | |
