# Supplementary material for: Efficacy and Safety of Xueshuantong Injection on Acute Cerebral Infarction: Clinical Evidence and GRADE Assessment
Source: Front Pharmacol. 2020 Jul 2;11:822. doi: 10.3389/fphar.2020.00822 (PMC7345308; doi:10.3389/fphar.2020.00822)
Supplement: Supplementary file 10 [file Table_4.docx]

| **Table S4. Adverse events.** | | | | | | | |
| --- | --- | --- | --- | --- | --- | --- | --- |
| **Study** | **Reference** | **Intervention** | | | **Adverse events** | | **Management for patients with adverse events (AEs)** |
|  |  | **Treatment group**  **(XST combined with CTs)** | **Control**  **group** | **Duration/day** | **Experimental group** | **Control group** |  |
|  |  | **Dosage of XST/mg** |  |  |  |  |  |
| Li 2019 | Li, 2019 | 4ml | CTs | 14 | Two cases of minor gastrointestinal reactions; elevation of transaminase in two patients; four cases of gingival bleeding. | Four cases of minor gastrointestinal reactions; elevation of transaminase in two patients; three cases of gingival bleeding. | AEs were tolerable and did not affect treatment. |
| Zhang 2016 | Zhang et al., 2016 | 300mg | CTs | 14 | Two patients had abnormal liver function test results; one patient had mild headache. | One patient had abnormal liver function test result. | AEs were tolerable and did not affect treatment. |
| Jiao 2016 | Jiao et al., 2016 | 4ml | CTs | 14 | Four patients had minor gastrointestinal reactions; elevation of transaminase were observed in three patients; three cases of gingival bleeding. | Three patients had minor gastrointestinal reactions; elevation of transaminase were observed in three patients; two cases of gingival bleeding. | AEs were tolerable and did not affect treatment. |
| Zheng 2016 | Zheng, 2016 | 2-5ml | CTs | 30 | One patient occurred without specific symptom reported. | Two patients occurred without specific symptoms reported. | AEs were tolerable and did not affect treatment. |
| He 2015 | He et al., 2015 | 500mg | CTs | 28 | Facial fever in two patients. | Mild dizziness in three patients. | Completely disappeared after proper rest. |
| Zhang 2014 | Zhang, 2014 | 250mg | CTs | 14 | Three patients had headache; two patients had fever; one patient had bleeding. | Two patients had headache; one patient had fever; one patient had a hypersensitivity reaction. | After symptomatic treatment, the symptoms disappeared. |
| Liang 2012 | Liang, 2012 | 300mg | CTs | 14 | Fifteen patients occurred without specific symptom reported. | Twelve patients occurred without specific symptom reported. | AEs were tolerable and did not affect treatment. |
| **XST:** Xueshuantong injection; **CTs**: Conventional treatments: thrombolytic drugs, anticoagulant, antiplatelet, antihypertensive, statins, neuroprotective agents, collateral circulation drugs and lipid-lowering medications. | | | | | | | |
